# Endophytic and antagonistic *Bacillus amyloliquefaciens* 8SE-IF1-derived nanoparticles encumber phytopathogenic oomycetes, fungi, bacteria, and viruses with enhanced growth in tomato seedlings

**DOI:** 10.3389/fmicb.2025.1612335

**Published:** 2025-08-01

**Authors:** A. Mary Sharmila, Joy Michal Johnson, Saru Sara Sam, Deepa R. Chandran, B. Ajay, G. Heera, S. Sarada, Usha C. Thomas, Swapna Alex, N. V. Radhakrishanan

**Affiliations:** ^1^Department of Plant Pathology, College of Agriculture, Vellayani, Kerala Agricultural University, Thiruvananthapuram, Kerala, India; ^2^Department of Vegetable Science, College of Agriculture, Vellayani, Kerala Agricultural University, Thiruvananthapuram, Kerala, India; ^3^Instructional Farm, College of Agriculture, Vellayani, Kerala Agricultural University, Thiruvananthapuram, Kerala, India; ^4^Department of Molecular Biology and Biotechnology, College of Agriculture, Vellayani, Kerala Agricultural University, Thiruvananthapuram, Kerala, India

**Keywords:** *Bacillus amyloliquefaciens*, antimicrobial metabolites, green-synthesized nanoparticles, *Phytophthora nicotianae*, *Fusarium oxysporum f. sp. lycopersici*, *Ralstonia solanacearum*, tomato leaf curl New Delhi virus, plant growth promotion

## Abstract

Green synthesis of nanoparticles (Gs-NPs) of antimicrobial compounds from endophytic and antagonistic microbes is a novel strategy for managing plant diseases caused by different pathogens. The present study aims to green synthesize the NPs of water-diffusible antimicrobial metabolites (WDM) from the antagonistic and endophytic bacterial strain *Bacillus amyloliquefaciens* 8SE-IF1 (*Ba*-8SE-IF1) against phytopathogenic oomycetes, fungi, bacteria, and viruses infecting tomato plants. The water-diffusible extract (WDE) of *Ba*-8SE-IF1 significantly inhibited the mycelial growth of *Pythium aphanidermatum* (44.6%), *Phytophthora nicotianae* (60.1%), *Fusarium oxysporum* f. sp. *lycopersici* (65.5%), and *Colletotrichum gloeosporioides* (66.7%) in poisoned food assays; the growth of *Ralstonia solanacearum* and *Xanthomonas campestris* in the agar well method (inhibition zones of 20.25 mm and 28.52 mm, respectively); and decreased the symptoms produced by tomato spotted wilt virus (TSWV) in local lesion host (68.3%) and tomato leaf curl New Delhi virus (ToLCNDV) in tomato plants (66.1%). Gas Chromatography-Mass Spectrometry-Mass Spectrometry (GC-MS/MS) analysis of the WDE of *Ba-*8SE-IF1 identified 26 major organic compounds with antimicrobial properties. Five compounds, *viz*. phenol 3,5-bis (1,1-dimethyl-ethyl), hexadecane, 1-tetradecene, 2,6,10,14-tetramethyl hexadecane, and 2,6,11,15-tetramethyl hexadecane, exhibited simultaneous antioomycete, antifungal, antibacterial, and antiviral activities. The identified antimicrobial compounds were phenols, carboxylic acids, alcohols, carbonyls of aldehydes, and aliphatic hydrocarbons. The stable, crystalline, and functional zinc oxide nanoparticles of *Ba*-8SE-IF1-water diffusible metabolites with a size of approximately 60 nm were green synthesized (Gs-ZnO-NPs-*Ba*-8SE-IF1-WDM). Gs-NPs, even at 100 ppm, drastically reduced the growth of *P. aphanidermatum* (43.1%), *P. nicotianae* (62.7%), *F. oxysporum* f. sp. *lycopersici* (84.6%), *C. gloeosporioides* (81.7%), *R. solanacearum* (21.24 mm), and *X. campestris* (18.92 mm); and the symptoms produced by TSWV (69.9%) and ToLCNDV (62.6%). Gs-NPs at 100 ppm significantly reduced the incidence of bacterial wilt caused by *R. solanacearum* to < 10% compared to more than 60% in control plants. Additionally, Gs-NPs considerably promoted plant height, number of branches and leaves, leaf area, and shoot and root biomass. To the best of our knowledge, this is the first study demonstrating the potential of *Ba*-8SE-IF1 and its WDE and Gs-ZnO-NPs-WDM for the simultaneous control of phytopathogenic oomycetes, fungal, bacterial, and viral diseases with enhanced growth traits in tomato plants.

## 1 Introduction

Plant diseases are commonly managed using crop protection chemicals, including fungicides, antibiotics, and insecticides. Excessive use of these chemicals adversely affects soil health, the environment, and human health. Utilizing crop protection chemicals at the nanoscale level addresses these issues. Nanotechnology is a multidisciplinary field with broad applications in the fields of science and technology. Silver (Ag) nanoparticles were the first to be investigated for plant disease management. Studies by Park et al. (2016), Lamsal et al. (2011), and Kim et al. (2008) examined the early use of silver nanoparticles in controlling powdery mildew disease in various crops. However, chemically synthesized nanoparticles can increase particle reactivity and toxicity, which may lead to negative effects on plant and human health and the environment through the breakdown of chemical groups and the formation of by-products (Meena et al., 2021).

Many biocontrol agents are widely used for the management of fungal and bacterial diseases, as these agents produce antifungal and antibacterial metabolites, which, in turn, control plant diseases (Vinodkumar et al., 2018; Basavarajappa et al., 2023). However, most biocontrol agents are non-specific and adversely affect beneficial microbes present in the soil. Beneficial endophytic and antagonistic microorganisms have been widely explored in the management of crop diseases (Johnson et al., 2014; Gill et al., 2016). The promising endophytic and antagonistic fungi or bacteria produce antimicrobial metabolites having either antifungal or antibacterial properties (Vinodkumar et al., 2018). Green synthesis of nanoparticles of antimicrobial compounds derived from potential endophytic and antagonistic microbes offers a sustainable approach for managing plant diseases because they are non-toxic, environmentally safe, effective at low dosage, target multiple fungal or bacterial pathogens, and safe for the beneficial organisms in the crop niche.

Tomato (*Lycopersicon esculentum* Mill.) is a widely grown vegetable that is rich in vitamins A, E, and C, as well as calcium, niacin, and organic acids, and has a high water content (Aslam et al., 2017). Due to their short growth period and high profitability, tomatoes are commercially used in the production of various food products (Karthika et al., 2020). As a result, there is an increasing demand for improving production techniques, fruit quality, yield, storage methods, and effective disease and pest management practices. Tomatoes are highly susceptible to various oomycetes, fungal, bacterial, and viral diseases, resulting in a substantial yield reduction and decreased nutritional value. Common fungal diseases affecting tomato cultivation include damping off, Phytophthora root rot, Fusarium wilt, and anthracnose (Singh et al., 2017). Among bacterial diseases, wilt and spot are particularly destructive, accounting for approximately 90% of the yield losses (Huang et al., 2013). Additionally, tomato spotted wilt virus and tomato leaf curl New Delhi virus pose a high risk to tomatoes, with yield losses ranging from 70 to 95% (Ong et al., 2020). The above diseases are managed by the regular use of fungicides or antibiotics. Insecticides are used to control vectors that transmit viral diseases. Moreover, the development of fungicide and antibiotic resistance adds further challenges to management. Therefore, nanopesticides offer an advanced solution for controlling various diseases in crop plants, as the quantity required is low.

Among metal nanoparticles, zinc nanomaterials are cost-effective, less phytotoxic, environmentally safe, and exhibit antimicrobial activity against phytopathogenic fungi (Yehia and Ahmed, 2013; Zabrieski et al., 2015; De La Rosa-García et al., 2018; Dos Santos et al., 2019; Kumawat et al., 2025), bacteria (Almoudi et al., 2018; Khan and Siddiqui, 2018; Rashid et al., 2024), viruses (Cai et al., 2019), and algae (Qureshi et al., 2018). Moreover, zinc is a crucial micronutrient for plant growth, carbohydrate metabolism, and the regulation of gene expression linked to biotic and abiotic stresses (Sabir et al., 2014). Therefore, zinc oxide nanoparticles have gained growing interest in agriculture because they are recognized as safe by the United States Food and Drug Administration (USFDA; FDA, 2015).

Green synthesis of nanoparticles can be performed using derivatives of plants as well as microorganisms such as fungi, bacteria, actinobacteria, yeasts, molds, and algae. Biomolecules found in plants or microorganisms, such as proteins, enzymes, phenolic compounds, amines, alkaloids, and pigments, serve as reducing agents in the synthesis of nanoparticles (Nadaroglu et al., 2017). In recent years, endophytes have gained importance in sustainable agriculture because of their unique ability to colonize plant tissues without causing disease. Besides being non-pathogenic, endophytes promote plant health and protect plants from biotic and abiotic stresses through the production of bioactive metabolites, improvement of nutrient availability, and modulation of the plant immune responses (Johnson et al., 2018, 2019; Khan et al., 2025). Endophytic bacteria are widely used for nanoparticle synthesis because of their ability to reduce metal toxicity (Korbekandi et al., 2009). They transform metals into nanoparticles through the activity of cellular enzymes and secondary metabolites (Joshi et al., 2017; Meena et al., 2021). This study highlights the simultaneous broad-spectrum antimicrobial activity of WDE and Gs-ZnO-NPs of the promising endophytic and antagonistic *Ba*-8SE-IF1 against tomato pathogens, including *P. aphanidermatum* (Edson), *P. nicotianae* (Breda de Haan), *C. gloeosporioides* (Penzig.), *F. oxysporum* f. sp. *lycopersici* (Sacc.), *R. solanacearum* (Smith), *X. campestris* (Dowson), TSWV, and ToLCNDV. GC-MS/MS analysis of the WDE of *Ba-*8SE-IF1 identified 26 major organic compounds with antioomycetes, antifungal, antibacterial, and antiviral properties. Gs-ZnO-NPs of *Ba*-8SE-IF1-WDM significantly inhibited the above pathogens, and the endophyte promotes the growth of tomato seedlings.

## 2 Materials and methods

### 2.1 Isolation, culture, and maintenance of *Ba*-8SE-IF1

Endophytic bacteria were isolated from the shoot and root tissues of tomato plants from various agro-ecological units in Kerala, India, following the procedure of Safdarpour and Khodakaramian (2017) with modifications. The plant samples were washed thoroughly under running tap water and cut into 0.5 cm segments. These segments were surface-sterilized with 0.1% mercuric chloride for 1 min, 4% sodium hypochlorite for 6 min, 70% ethanol for 2 min, and finally rinsed three times with sterile double-distilled (dd) water. Aliquots of the final rinse water were inoculated in tryptic soy broth (TSB; casein peptone, 15 g; soybean peptone, 5 g; NaCl, 5 g; dd water, 1 L; pH 7.5) and incubated at 27 ± 2°C and relative humidity (RH) of 80 ± 5% for 72 h to ensure the absence of turbidity due to contamination by surface-living bacteria. The surface-sterilized plant tissues were then ground using a sterile mortar and pestle in 3 mL of sodium phosphate buffer (0.01 M, pH 7.0). Serial dilutions were prepared from the resulting extract, and aliquots from dilutions ranging from 10^−1^ to 10^−3^ were spread onto TSA plates. These plates were incubated at 27 ± 2°C and RH 80 ± 5%. Subsequently, bacterial colonies were selected based on their growth characteristics at 3, 5, and 7 days after incubation (DAI). The isolated endophytic bacteria were screened against *P. aphanidermatum, P. nicotianae, F. oxysporum* f. sp. *lycopersici, C. gloeosporioides, R. solanacearum, X. campestris*, TSWV, and ToLCNDV (paper communicated). *Ba*-8SE-IF1 (the 16S rRNA sequence was submitted to NCBI GenBank with the accession number PV023912) exhibited the highest antimicrobial activity against phytopathogens and was also endophytic in tomato and other crop plants (paper communicated). A pure culture of *Ba*-8SE-IF1 was maintained in nutrient agar (NA; peptone, 5 g; beef extract, 2.5 g; NaCl, 5 g; agar, 10 g; dd water, 1 L) and broth (NB) media (pH 6.8) at 27 ± 2°C and RH 80 ± 5% for further studies.

### 2.2 Extraction of WDE of *Ba*-8SE-IF1

*Ba*-8SE-IF1 was cultured in NB medium and incubated at 27 ± 2°C and RH 80 ± 5% for 48 h in a shaking incubator (REMI Elektrotechnik Ltd., India) at 120 rpm. After 48 h, the maximum bacterial population (4 × 10^8^ CFU/mL) was assessed with an optical density (OD_600_) value of 0.8 (LAMBDA 365 UV-Vis Spectrophotometer—PerkinElmer, USA) and also with serial dilution followed by spread plating. The bacterial cells as pellets were collected by centrifuging the bacterial broth at 3,000 rpm for 10 min using a centrifuge (Eppendorf, Germany). The collected pellet was washed twice with sterile double-distilled water and centrifuged at 3,000 rpm for 5 min. The bacterial pellets were used for the extraction of the WDE. For this, the bacterial pellets were resuspended in sterile double-distilled water at a concentration of 10^10^ CFU/mL and incubated at 27 ± 2°C and RH 80 ± 5% for 48 h in a shaking incubator at 120 rpm. After the incubation period, the supernatant containing water-diffusible metabolites was separated by centrifugation at 5,000 rpm for 15 min, discarding the pellet, thus forming the WDE. The WDE was concentrated to 1/10^th^ volume by evaporation in a rotary flask evaporator (LabTech, Italy) at 42°C and 50 rpm and was used to assess the antimicrobial properties.

### 2.3 Evaluation of WDE-*Ba*-8SE-IF1 against phytopathogenic oomycetes, fungi, bacteria, and viruses

#### 2.3.1 Cultures and maintenance of the oomycetes, fungi, bacteria, and viruses

Cultures of phytopathogenic oomycetes (*P. aphanidermatum* and *P. nicotianae*) and fungi (*F. oxysporum* f. sp. *lycopersici* and *C. gloeosporioides*) were isolated from tomato plants exhibiting characteristic symptoms and pure cultured. The pathogenicity of these isolates was confirmed by Koch's postulates, and the cultures were maintained in potato dextrose agar (PDA; peeled potato, 200 g; dextrose, 20 g; agar, 10 g; dd water, 1 L) medium (pH 6.5) at 27 ± 2°C and RH 80 ± 5% at the Department of Plant Pathology, College of Agriculture, Vellayani, Kerala Agricultural University (KAU), India.

The bacterium *R. solanacearum* was isolated from bacterial wilt-infected tomato plants using triphenyl tetrazolium chloride medium (TTC; pH 6.8). Bacterial ooze from the infected plant samples was collected in sterile dd water, and 50 μL of the oozed suspension was plated and spread uniformly onto the medium. The inoculated plates were then incubated for 48 h at 27 ± 2°C and RH 80 ± 5%. Highly fluidal, irregular, large colonies with a pink hue surrounded by a creamy white border were maintained on TTC medium (peptone, 10 g; casein hydrolysate, 1 g; glucose, 5 g; agar, 10 g; 5 mL of 1% 2,3,5-triphenyl tetrazolium chloride; dd water, 1 L) for further studies. The 16S rRNA sequence of *R. solanacearum* was submitted to the NCBI GenBank with the accession number PV022497. *X. campestris* was isolated in NA medium from bacterial spot-infected tomato plants, and the pure culture was preserved at the department. These phytopathogenic bacteria were subcultured on nutrient agar medium at 27 ± 2°C and RH 80 ± 5% for further studies. The virulence of the oomycetes, fungi, and bacteria was maintained by periodic inoculation, isolation, pure culturing, and subculturing.

TSWV and ToLCNDV were maintained in tomato plants (var. Vellayani Vijay released by KAU) by insect vectors or graft transmission following a standard protocol in a ventilated insect-proof glasshouse (Chandran et al., 2021; Sam, 2021). Individual plants showing typical symptoms of tomato spotted wilt and leaf curl were placed in insect-proof cages (50 cm × 50 cm × 50 cm), kept in a glasshouse, and maintained as the source of the virus inoculum for further studies.

#### 2.3.2 Evaluation of the WDE against the oomycetes and fungi by the poisoned food technique

PDA was supplemented with WDE at a ratio of 100:1 (v/v). Pathogens with 5 mm mycelial discs (7 days old) were cut from the growing edge of the colony using a sterile cork borer and placed in the center of the PDA plates amended with WDE. Five replications of the inoculated plates, along with the control (without WDE), were incubated at 27 ± 2°C until complete fungal growth was observed in the control plates. Radial mycelial growth of each fungus was measured separately, and the percentage inhibition was calculated using the following formula:


$$\begin{array}{l} \text{Percent inhibition }\left(\text{PI}\right)=\frac{\text{C}-\text{T}}{\text{C}}\times \;100 \end{array}$$

C: growth in control plates (cm); T: growth in treated plates (cm).

#### 2.3.3 Evaluation of the WDE against bacterial pathogens by the agar well method

*R. solanacearum* and *X. campestris* (each 50 μL; 10^4^ CFU/mL) were uniformly spread on separate NA plates. A single well of 7 mm diameter was made in each plate using a cork borer. Each well was filled with 30 μL of WDE with five replications. The inoculated plates were incubated at 27 ± 2°C for 48 h. Antagonistic efficacy of the WDE was determined by measuring the inhibition zone (mm) around the agar wells.

#### 2.3.4 Evaluation of the WDE against the viruses

##### 2.3.4.1 Tomato spotted wilt virus in the local lesion host

The local lesion host, *Chenopodium amaranticolor* (Coste and A. Reyn) plants, were grown in an insect-proof glasshouse for 1 month, and fully expanded leaves were used for the mechanical/sap transmission of TSWV. Leaves were smeared with WDE and allowed to dry. After 24 h, the sap of TSWV-infected leaves (extracted in 0.1 M potassium phosphate buffer (pH 7.0) containing 1 mL of β-mercapto-ethanol in 1 L buffer; 200 mg of infected tissue in 1 mL buffer) was gently swabbed after dusting carborundum powder on leaves. The inoculated plants were kept in an insect-proof glasshouse at 27 ± 2°C with an RH of 80 ± 5% for the development of symptoms such as lesions in five leaves per plant with five replications. Leaves inoculated with buffer alone were used as controls.

##### 2.3.4.2 Tomato leaf curl New Delhi virus in tomato plants

Tomato plants of the variety Vellayani Vijai (released by KAU), aged 30 days, grown in an insect-proof glasshouse, were sprayed with the WDE. After 24 h, the ToLCNDV-infected scion was grafted onto the treated plant for transmission of the virus and development of the characteristic symptoms. The inoculated plants were kept in an insect-proof glasshouse at 27 ± 2°C with an RH of 80 ± 5%, with five replications. Plants sprayed with buffer alone served as the control. The severity of the disease was assessed as per Bos (1982).

### 2.4 Analysis of antimicrobial compounds in WDE-*Ba*-8SE-IF1 through GC-MS/MS

The antimicrobial metabolites in the WDE were identified by GC-MS/MS using a Thermo GC-Trace Ultra (Version 5.0) and Thermo MS DSQ II (Thermo Fisher Scientific, USA). The equipment had a DB-35-MS capillary standard non-polar column with dimensions of 30 mm × 0.25 mm ID × 0.25 μm film. The carrier gas used was helium at a flow of 1.0 mL/min. The injector was operated at 250°C, and the oven temperature was programmed as follows: 60°C for 15 min, then gradually increased to 280°C for 3 min. The compounds were identified by referencing the Wiley and NIST libraries and by comparing their retention indices provided by the GC-MS instrument.

### 2.5 Green synthesis of zinc oxide nanoparticles of water-diffusible metabolites of *Ba*-8SE-IF1

The green synthesis of nanoparticles of WDM of *Ba-*8SE-IF1 on zinc oxide was done as per the protocol of Iqtedar et al. (2020) with modifications. A solution of zinc sulfate heptahydrate (0.01 M) was mixed with WDM-*Ba*-8SE-IF1 (1/10^th^ volume concentrated) at a ratio of 1:1 to facilitate the reduction of zinc. The mixture was incubated at 37°C and 120 rpm for 48 h in a shaking incubator. After 48 h, a color change was observed. The color-changed nanoparticle solutions were centrifuged at 10,000 rpm for 30 min, and the resulting pellets were resuspended in deionized water to eliminate biological contaminants and centrifuged again. The pellets were dried in a hot-air oven at 40°C and stored at 4°C for further characterization and *in vitro* studies.

#### 2.5.1 Characterization of green-synthesized nanoparticles

The formation of green-synthesized nanoparticles was initially confirmed using a LAMBDA 365 UV-Vis spectrophotometer. Absorption measurements were recorded in the wavelength range of 200–600 nm. A particle size analyzer (PerkinElmer, USA) was used to determine the size of the nanoparticles capped with biomolecules and measure their stability. Fourier Transform Infrared Spectroscopy (FTIR; PerkinElmer, USA) was used to identify the antimicrobial metabolites involved in the metal reduction process, with spectral data collected in the range of 400–4,000 cm^−1^ at room temperature. The crystalline pattern of synthesized nanoparticles was further analyzed using an X-ray diffraction (XRD; Thermo Fisher Scientific, USA) technique, and the 2Θ range was recorded between 20 and 80° with a scanning speed of 6 min^−1^. The morphology of the green-synthesized nanoparticles was examined using a Field Emission Scanning Electron Microscope (FE-SEM; Hitachi High-Tech Corporation, Japan).

### 2.6 Evaluation of the Gs-NPs against phytopathogenic oomycetes, fungi, bacteria, and viruses

Green-synthesized zinc oxide nanoparticles of WDM-*Ba*-8SE-IF1 (100 ppm) were evaluated against *P. aphanidermatum, P. nicotianae, F. oxysporum* f. sp. *lycopersici*, and *C. gloeosporioides* by the poisoned food technique; *R. solanacearum* and *X. campestris* through the agar well method; and TSWV by local lesion assay and ToLCNDV on tomato plants as described in Section 2.3.

### 2.7 *In vivo* evaluation of green-synthesized nanoparticles against *R. solanacearum*

The efficacy of green-synthesized nanoparticles was evaluated against *R. solanacearum in vivo*. Tomato seedlings of var. Pusa Ruby (released by ICAR-Indian Agricultural Research Institute, New Delhi, India) were grown in pots filled with a mixture of sand, soil, and farmyard manure (1:1:1), as per the Packages of Practices Recommendations of KAU (KAU, 2024). To assess the efficacy of NPs against *R. solanacearum* in tomato plants, Gs-NPs were foliar-sprayed and soil-drenched at 100 ppm in 4-week-old tomato plants. After 5 days, *R. solanacearum* grown in TTC broth was artificially inoculated by soil drenching to the plants at 4 × 10^8^ CFU/mL (OD_600_ value of 0.8). The details of the treatments included absolute control, *R. solanacearum* alone at 10^8^ CFU/mL (positive control), Gs-NPs alone at 100 ppm, and Gs-NPs + *R. solanacearum*. Observations on the incidence of bacterial wilt disease at 10 days after treatment and biometric parameters, *viz.*, plant height, number of branches, number of leaves, leaf area, and shoot and root biomass, were taken from 10 plants per treatment at 20 days after treatment. Bacterial wilt incidence was calculated by the formula


$$\begin{array}{l} \text{Disease incidence }\left(\text{\%}\right)=\frac{\text{Number of infected plants}}{\text{Total number of plants}}\times 100 \end{array}$$

### 2.8 Statistical analysis

All experiments were done using a completely randomized design (CRD) with a minimum of five replications. The data were analyzed using the statistical software GRAPES, developed by Kerala Agricultural University (Gopinath et al., 2021), with a 5% level of significance.

## 3 Results

### 3.1 Cultural characteristics of the most promising endophytic and antagonistic bacterium, *B. amyloliquefaciens* 8SE-IF1

The cultural and morphological characteristics, *viz*., colony color, form, margin, texture, and Gram staining, were studied according to Bergey's Manual of Determinative Bacteriology. The colony characteristics of the most promising endophytic and antagonistic bacterial strain, *B. amyloliquefaciens* 8SE-IF1, on NA medium were dull white, medium-sized, irregularly bordered with wavy and undulate margins, and slimy mucoid texture (Supplementary Figure S1a). Gram staining of the endophytic bacterial strain indicated its Gram-positive (G^+^) nature. In NB broth, *Ba*-8SE-IF1 was typically pale yellowish and turbid and formed a thin, off-white film at the air-liquid interface due to its aerobic nature (Supplementary Figure S1b).

### 3.2 Water-diffusible extract of *Ba*-8SE-IF1 inhibited the growth of phytopathogenic oomycetes, fungi, bacteria, and viruses infecting tomato plants

**Figure 1:**
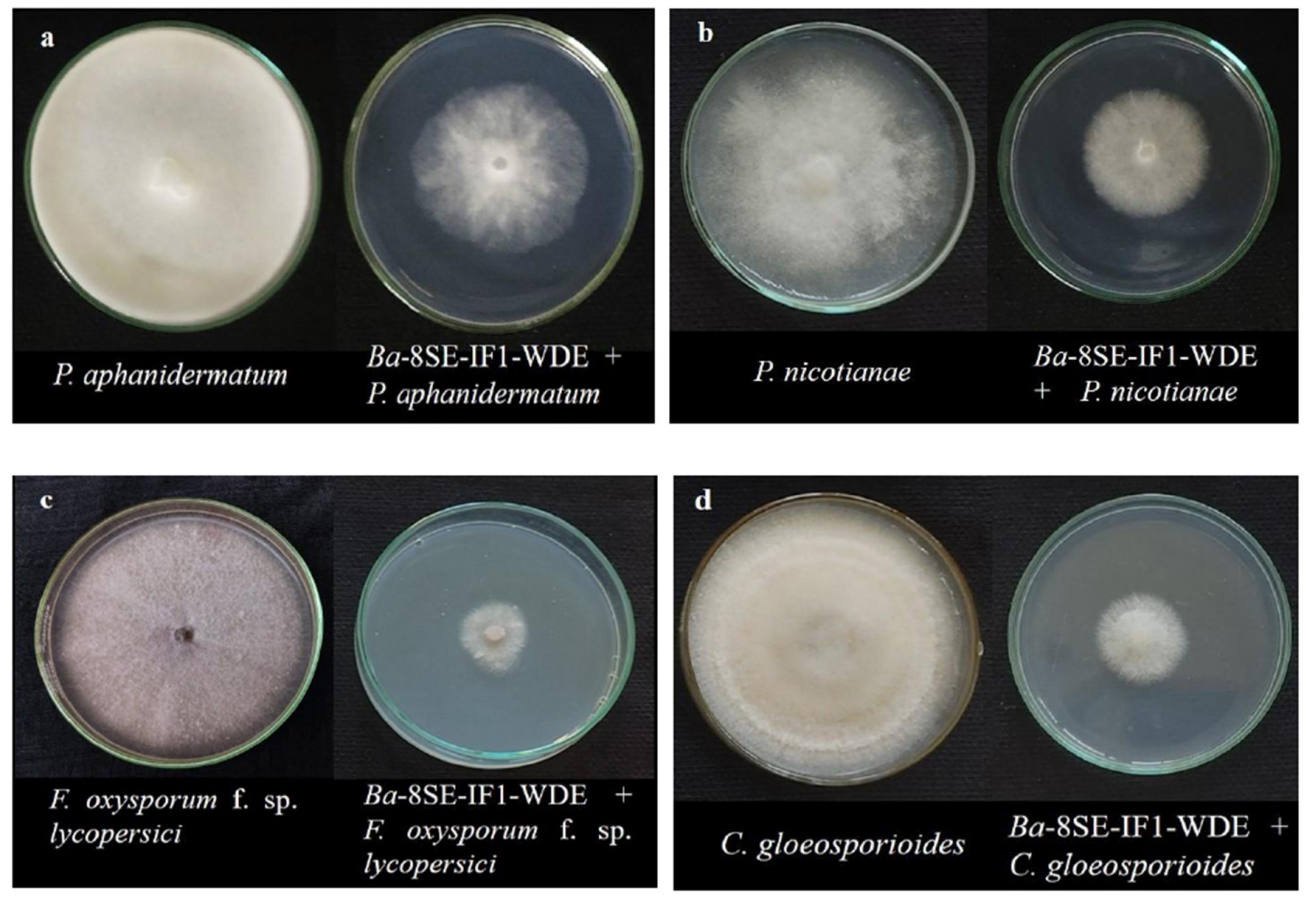
Effect of water diffusible extract of *Bacillus amyloliquefaciens* 8SE-IF1 on phytopathogenic oomycetes infecting tomato plants **(a)** *P. aphanidermatum;* **(b)** *Phytophthora nicotianae*; and fungi infecting tomato plants **(c)** *Fusarium oxysporum* f. sp. *lycopersici* and **(d)** *Colletotrichum gloeosporioides*. Representative pictures from five independent experiments.

**Figure 2:**
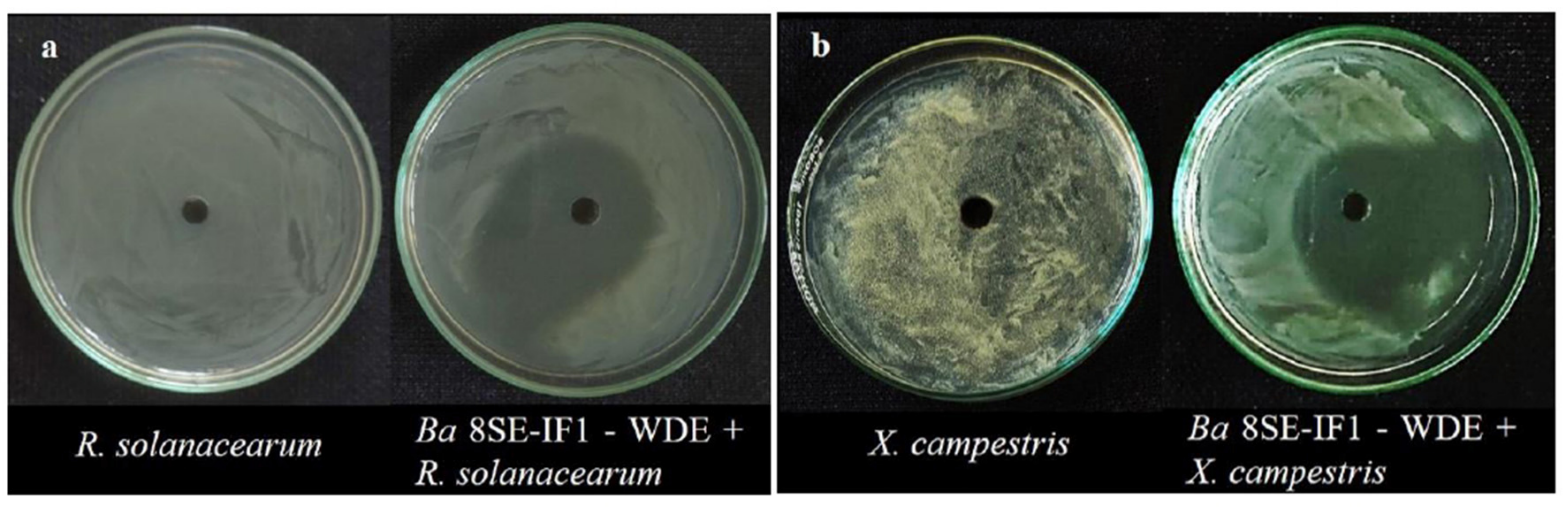
Effect of water diffusible extract of *Bacillus amyloliquefaciens* 8SE-IF1 on phytopathogenic bacteria infecting tomato plants **(a)** *Ralstonia solanacearum* and **(b)** *Xanthomonas campestris*. Representative pictures from five independent experiments.

**Figure 3:**
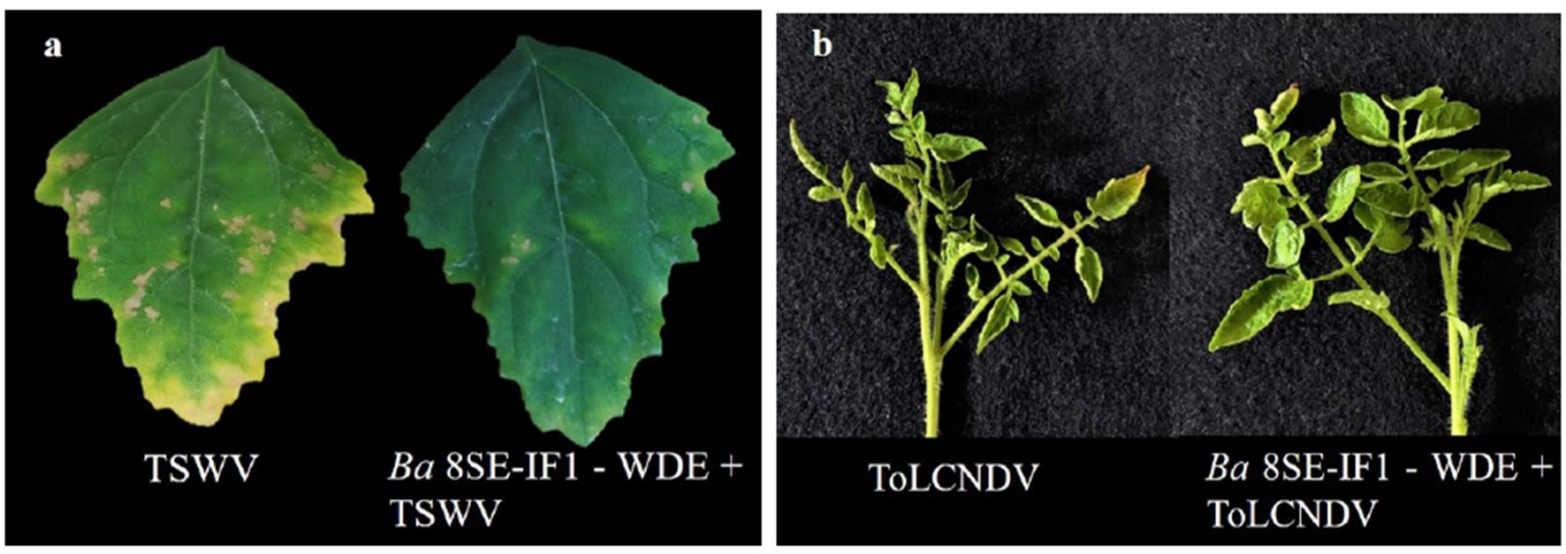
Effect of water diffusible extract of *Bacillus amyloliquefaciens* 8SE-IF1 against viruses infecting tomato plants **(a)** TSWV - Tomato spotted wilt virus symptoms on local lesion host, *C. amaranticolor* and **(b)** ToLCNDV - Tomato leaf curl New Delhi virus symptoms on systemic host, tomato. Representative pictures from three independent experiments.

The antimicrobial activity of the WDE of *Ba*-8SE-IF1 was evaluated against phytopathogenic oomycetes and fungi using the poisoned food technique, bacteria by the agar well method, and viruses by sap and graft transmission. The WDE significantly inhibited the mycelial growth of *P. aphanidermatum, P. nicotianae, F. oxysporum* f. sp. *lycopersici*, and *C. gloeosporioides*; growth of *R. solanacearum* and *X. campestris*; and decreased the symptoms produced by TSWV and ToLCNDV (Table 1; Figures 1–3). The highest fungal inhibition of 66.7% was observed against *C. gloeosporioides*, followed by *F. oxysporum* f. sp. *lycopersici* (65.5%) and *P. nicotianae* (60.1%; Table 1A; Figure 1). The lowest antifungal effect was recorded against *P. aphanidermatum* with an inhibition of 44.6%. Similarly, the highest antibacterial activity was recorded against *X. campestris*, which exhibited an inhibition zone of 28.52 mm, followed by *R. solanacearum*, with an inhibition zone of 20.25 mm (Table 1B; Figure 2). Similarly, foliar application of WDE significantly reduced the number of local lesions produced by TSWV in *C. amaranticolor* with a percent inhibition of 68.3, and tomato leaf curl disease severity assessed as a vulnerability index due to ToLCNDV with a percent inhibition of 66.1% (Tables 1C, D; Figure 3). Therefore, the WDE of the promising endophytic and antagonistic *Ba*-8SE-IF1 has the potential antimicrobial properties against different oomycetes, fungi, bacteria, and viruses that infect tomato plants.

**Table 1:** Effect of water-diffusible extract of promising endophytic and antagonistic bacterial strain, *B. amyloliquefaciens* 8SE-IF1, against different phytopathogenic oomycetes, fungi, bacteria, and viruses infecting tomato plants.

**A**	**Radial mycelial growth (cm)**	**Percent inhibition**
*Ba*-8SE-IF1-WDE *+ P. aphanidermatum*	4.98 ± 0.11^b^	44.6
*Ba*-8SE-IF1-WDE *+ P. nicotianae*	3.59 ± 0.33^c^	60.1
*Ba*-8SE-IF1-WDE + *F. oxysporum* f. sp. *lycopersici*	3.10 ± 0.07^d^	65.5
*Ba*-8SE-IF1-WDE *+ C. gloeosporioides*	2.99 ± 0.20^e^	66.7
Control	9.00 ± 0.00^a^	-
SE (m)	0.110	-
CD (0.05)	4.068	-
**B**	**Inhibition zone (mm)**	**-**
*Ba*-8SE-IF1-WDE *+ R. solanacearum*	20.25 ± 0.12^b^	-
*Ba*-8SE-IF1-WDE *+ X. campestris*	28.52 ± 0.62^a^	-
Control	0.0 ± 0.00^c^	-
SE (m)	0.280	-
CD (0.05)	2.428	-
**C**	**Number of lesions**	**Percent inhibition**
*Ba*-8SE-IF1-WDE + TSWV	5.23 ± 0.21^b^	68.3
Control	16.51 ± 0.82^a^	-
SE (m)	0.845	-
CD (0.05)	1.015	-
**D**	**Vulnerability index**	**Percent inhibition**
*Ba*-8SE-IF1-WDE + ToLCNDV	22.41 ± 0.54^b^	66.1
Control	66.20 ± 0.75^a^	-
SE (m)	0.624	-
CD (0.05)	0.968	-

A: Oomycetes and fungal pathogens; B: Phytopathogenic bacteria; C: TSWV: tomato spotted wilt virus and D: ToLCNDV: tomato leaf curl New Delhi virus; Values are the mean of five replications ± standard deviation; SE, Standard error; CD, Critical difference (0.05); Superscripts with the same alphabets indicate on-par values, and those in different alphabets indicate a significant difference at the 5% level of significance.

### 3.3 Antifungal, antibacterial, antiviral, and antimicrobial compounds were identified in the WDE of *Ba*-8SE-IF1 through GC-MS/MS

**Figure 4:**
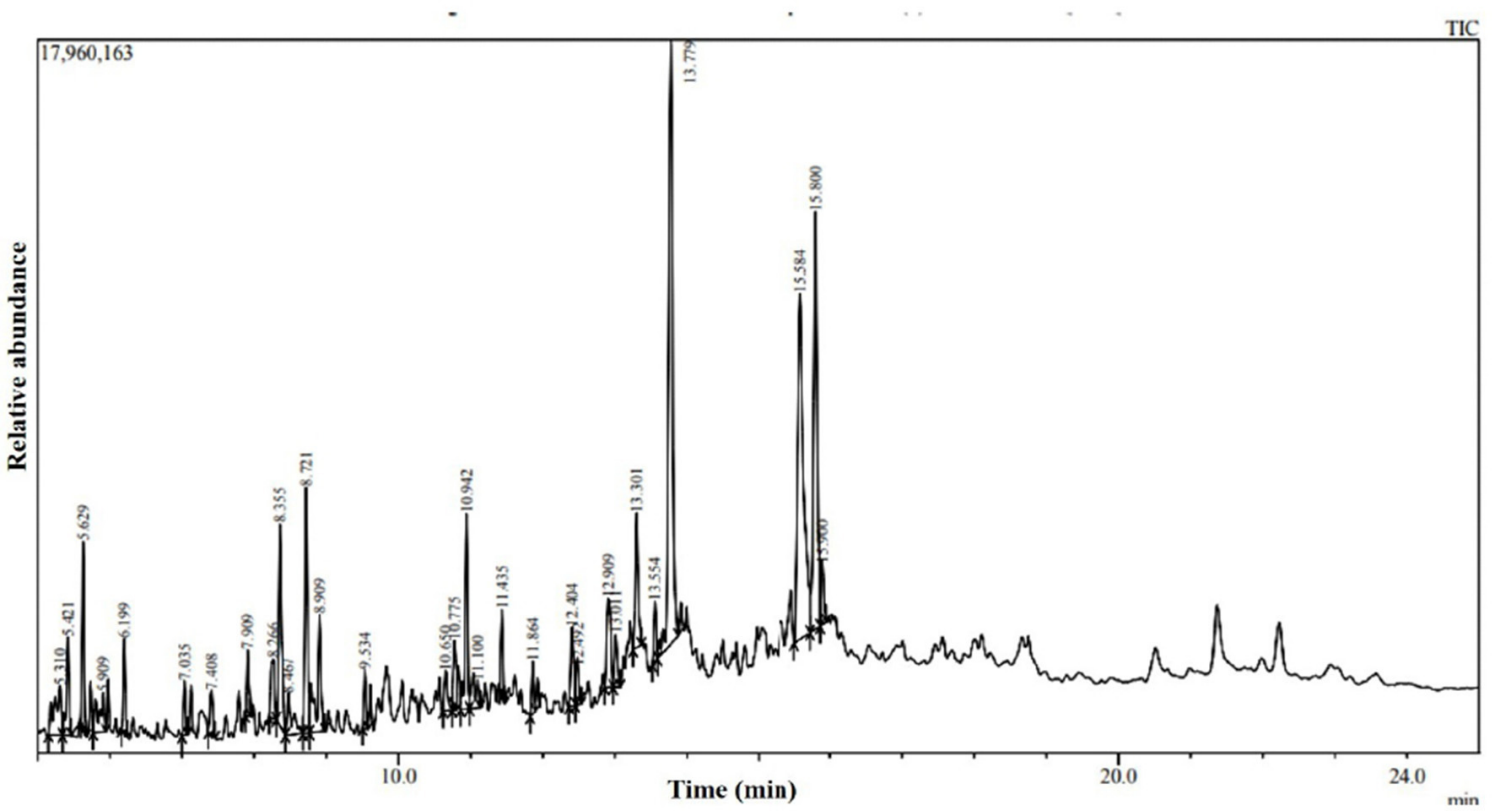
GC-MS/MS chromatogram of water diffusible extract of *Bacillus amyloliquefaciens* 8SE-IF1.

**Figure 5:**
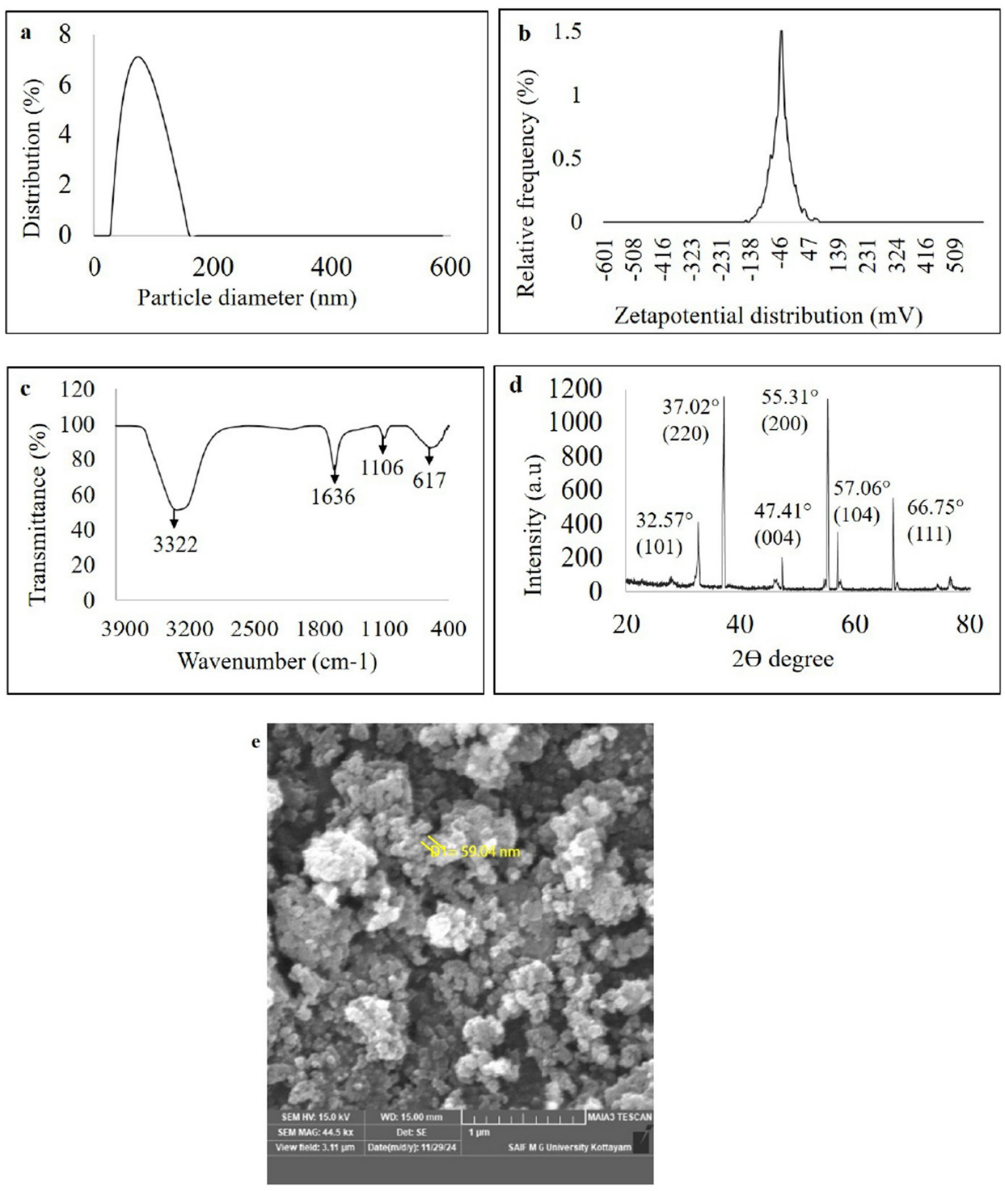
Characterization of green synthesized nanoparticles of WDM of *B. amyloliquefaciens* 8SE-IF1 on zinc oxide **(a)** Nano-particle size determination using dynamic light scattering; **(b)** Stability of nano- particles through zeta potential analysis; **(c)** Fourier transform infrared spectrum (FTIR) analysis; **(d)** Crystalline nature of nanoparticles through X ray diffraction (XRD); and **(e)** Morphology and size of green synthesized nanoparticles by Field emission Scanning electron microscope (FE-SEM).

The water-diffusible antimicrobial metabolites produced by the most promising endophytic bacterial strain, *Ba-*8SE-IF1, were analyzed using GC-MS/MS (Figure 4). Interestingly, a total of 26 major compounds were identified with either antifungal, antibacterial, antiviral, or antimicrobial activities (Table 2). Chemical name and its retention time, peak area percentage, and mass spectrum of the compounds having antifungal, antibacterial, antiviral, and antimicrobial activities are detailed in Table 2, Figure 5, and Supplementary Figure S2.

**Table 2:** Antimicrobial compounds identified in water-diffusible extract of *B. amyloliquefaciens* 8SE-IF1 through GC-MS/MS.

**Sl. No**.	**Chemical name**	**Functional groups**	**Retention time (min)**	**Peak area percentage**	**Biological properties**	**References**
1	Oleic acid	Phenol and carboxylic acid derivatives	15.58	15.54	Antifungal	Walters et al., 2004
2	Hexadecanoic acid		13.77	18.69	Antifungal	Sathyaprabha et al., 2010
3	Octadecanoic acid		12.40	1.99	Antifungal	Dheepa et al., 2016
4	1,2-Benzenedicarboxylic acid		12.90	2.84	Antifungal	Duan et al., 2021
5	Phenol, 3,5-bis (1,1-dimethyl-ethyl)		8.72	5.35	Antifungal, antibacterial, antiviral	Dharni et al., 2014; Rice et al., 2019
6	n-Nonadecanol-1	Alcohols and carbonyl group (aldehyde) derivatives	11.86	0.91	Antifungal	Faridha Begum et al., 2016
7	Iron, tricarbonyl[N-(phenyl-2-pyridinylmethylene) benzenamine-N,N']		11.43	1.27	Antifungal	Abdel-Hafez et al., 2015
8	2-butyl 1-Octanol		10.77	1.58	Antifungal	Mannaa and Kim, 2018
9	1-Hexadecanol		9.53	0.92	Antibacterial	Chatterjee et al., 2018
10	4,6-dimethyl dodecane	Aliphatic hydrocarbon derivatives (alkanes)	5.31	2.47	Antibacterial	Togashi et al., 2007
11	5-methyl tetradecane		5.42	2.10	Antibacterial	Rahbar et al., 2012
12	Tricosane		5.62	2.92	Antifungal	Basavarajappa et al., 2023
13	3,8-dimethyl undecane		5.90	1.65	Antifungal	Fernando et al., 2005
14	Hexadecane		6.19	1.43	Antifungal, antibacterial, antiviral	Yogeswari et al., 2012
15	1-Tetradecene		7.03	0.83	Antifungal, antibacterial, antiviral	Girija et al., 2014
16	9-methyl nonadecane		7.40	0.90	Antifungal	Prakash and Arora, 2021
17	2,6,10,14-tetramethyl hexadecane		7.90	1.10	Antifungal, antibacterial, antiviral	Yogeswari et al., 2012
18	2,6,10,15-tetramethyl heptadecane		8.26	1.63	Antibacterial	Rahbar et al., 2012
19	Octacosane		8.35	4.50	Antifungal	Awan et al., 2023
20	Non-adecane		8.46	1.61	Antifungal	Prakash and Arora, 2021
21	Heptadecane		8.90	3.82	Antifungal	Prakash and Arora, 2021
22	2,6,11,15-tetramethyl hexadecane		10.94	3.87	Antifungal, antibacterial, antiviral	Yogeswari et al., 2012
23	2-methyl octacosane		11.10	1.34	Antifungal	Awan et al., 2023
24	Heneicosane		12.49	0.99	Antibacterial	UshaNandhini et al., 2015
25	n-Tetradecane		13.30	2.80	Antibacterial	Girija et al., 2014
26	Dotriacontane		15.90	1.42	Antifungal	Bordoloi et al., 2017

The identified compounds were categorized into various functional groups, including phenols, carboxylic acids, alcohols, carbonyl groups of aldehydes, and aliphatic hydrocarbons (alkanes; Table 2). Major compounds, such as oleic acid, hexadecanoic acid, octadecanoic acid, 1,2-benzene-dicarboxylic acid, phenol, and 3,5-bis (1,1-dimethylethyl), are classified under the O-H stretching functional groups of phenols and carboxylic acids and have antifungal, antibacterial, and antiviral properties. Similarly, compounds such as n-nonadecanol-1, iron tricarbonyl [N-(phenyl-2-pyridinylmethylene) benzenamine-N, N'], 2-butyl-1-octanol, and 1-hexadecanol are associated with the alcohol and carbonyl (C=O) groups of aldehydes and possess either antifungal or antibacterial activities (Table 2). The aliphatic hydrocarbons (alkanes), including 4,6-dimethyl dodecane, 5-methyl tetradecane, tricosane, 3,8-dimethyl undecane, hexadecane, 1-tetradecene, 9-methyl nonadecane, 2,6,10,14-tetramethyl hexadecane, 2,6,10,15-tetramethyl heptadecane, octacosane, nonadecane, heptadecane, 2,6,11,15-tetramethyl hexadecane, 2-methyl octacosane, heneicosane, tetradecane, and dotriacontane, are also have either antifungal, antibacterial, antiviral, or antimicrobial properties. Surprisingly, five compounds, *viz.*, phenol 3,5-bis (1,1-dimethyl-ethyl), hexadecane, 1-tetradecene, 2,6,10,14-tetramethyl hexadecane, and 2,6,11 and 15-tetramethyl hexadecane, exhibited antifungal, antibacterial, antiviral, and antimicrobial activities (Table 2). The identification of major functional groups, such as phenols, carboxylic acids, alcohols, and carbonyl groups of aldehydes, further supports the potential of these metabolites in the synthesis of nanoparticles.

### 3.4 Characteristics of green-synthesized ZnO-NPs using WDM of *Ba*-8SE-IF1

The ZnO-NPs were green-synthesized using *Ba*-8SE-IF1-WDM in combination with the precursor salt zinc sulfate heptahydrate (ZnSO4 .7H_2_O). The formation of Gs-ZnO-NPs was confirmed by the characteristic color change from light to dark brown. The green synthesis of nanoparticles was initially confirmed using a UV-Vis spectrophotometer. The absorption peak for green-synthesized zinc oxide nanoparticles (Gs-ZnO-NPs) was observed at 387 nm, which ensures the presence of nanoparticles in the solutions (Supplementary Figure S3). The size and stability of the Gs-ZnO-NPs were confirmed using a particle size analyzer. Particle size distribution analysis showed a single peak with 100% intensity, indicating that the Gs-ZnO-NPs with a capping agent had an average size of 60 nm, with a range of 45 to 75 nm (Figure 5a). Similarly, the stability of the nanoparticles was assessed through zeta potential measurements. Gs-ZnO-NPs demonstrated good stability with a zeta potential of −33.1 mV (Figure 5b).

The FTIR spectrum of the Gs-ZnO-NPs exhibited characteristic absorption bands at 3,322, 1,636, 1,106, and 617 cm^−1^ (Figure 5c). The strong band at 3,322 cm^−1^ was attributed to O–H stretching vibrations, indicating the presence of alcohol and phenolic groups. The peak at 1,636 cm^−1^ corresponds to carbonyl (C=O) stretching of the aldehyde groups. The absorption band at 1,106 cm^−1^ was attributed to C–O stretching, indicative of the presence of carboxylic acid. The band observed at 617 cm^−1^ is attributed to the vibrational frequency of oxide (–O) bonds, confirming the formation of ZnO nanoparticles. The results presented in section 3.3 further confirm the presence of phenols and carboxylic acid (O–H stretching) metabolites as well as alcohol and carbonyl (C=O) groups of aldehydes in the Gs-ZnO-NPs. FTIR analysis indicated that the functional groups present in the metabolites acted as capping agents.

The crystalline and amorphous properties of the green-synthesized nanoparticles were also analyzed using the XRD technique. The XRD results showed diffraction peaks for Gs-ZnO-NPs at 32.57°, 37.02°, 47.41°, 55.31°, 57.06°, and 66.75°, corresponding to the crystal planes (101), (220), (004), (200), (104), and (111), respectively. These sharp peaks confirm the crystalline nature of the Gs-ZnO-NPs (Figure 5d). The structural and morphological characteristics of the green-synthesized nanoparticles were examined by FE-SEM. The FE-SEM results showed that the Gs-ZnO-NPs exhibited an oval to spherical shape with a size of 59.94 nm (Figure 5e).

### 3.5 Green-synthesized ZnO-NPs of WDM from *Ba*-8SE-IF1 inhibited the growth of phytopathogens infecting tomato plants

**Figure 6:**
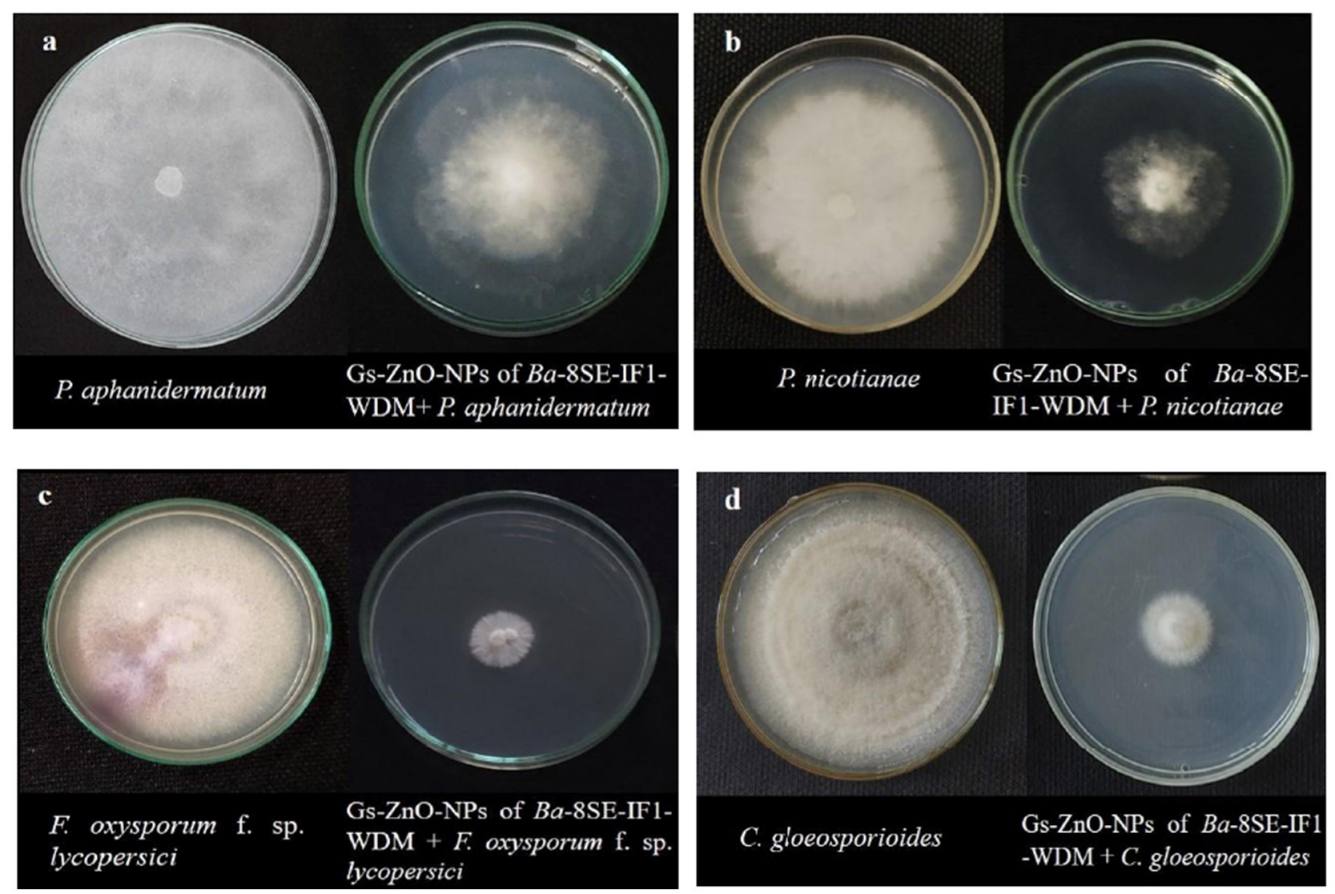
Effect of green synthesized ZnO nanoparticles of WDM of *Ba* 8SE-IF1 on phytopathogenic oomycetes infecting tomato plants **(a)** *P. aphanidermatum*; **(b)** *Phytophthora nicotianae*; and fungi infecting tomato plants **(c)** *Fusarium oxysporum* f. sp. *lycopersici* and **(d)** *Colletotrichum gloeosporioides*. Representative pictures from three independent experiments.

**Figure 7:**
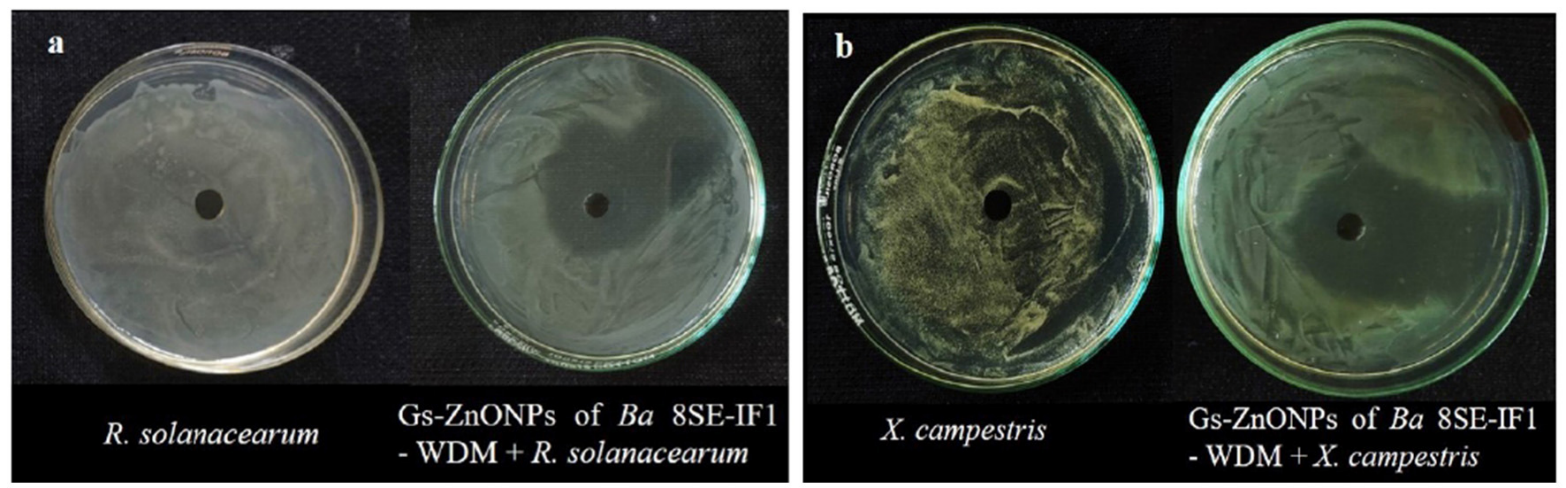
Effect of green synthesized ZnO nanoparticles of WDM of *Ba* 8SE-IF1 against phytopathogenic bacteria infecting tomato plants **(a)** *Ralstonia solanacearum* and **(b)** *Xanthomonas campestris*. Representative pictures from three independent experiments.

**Figure 8:**
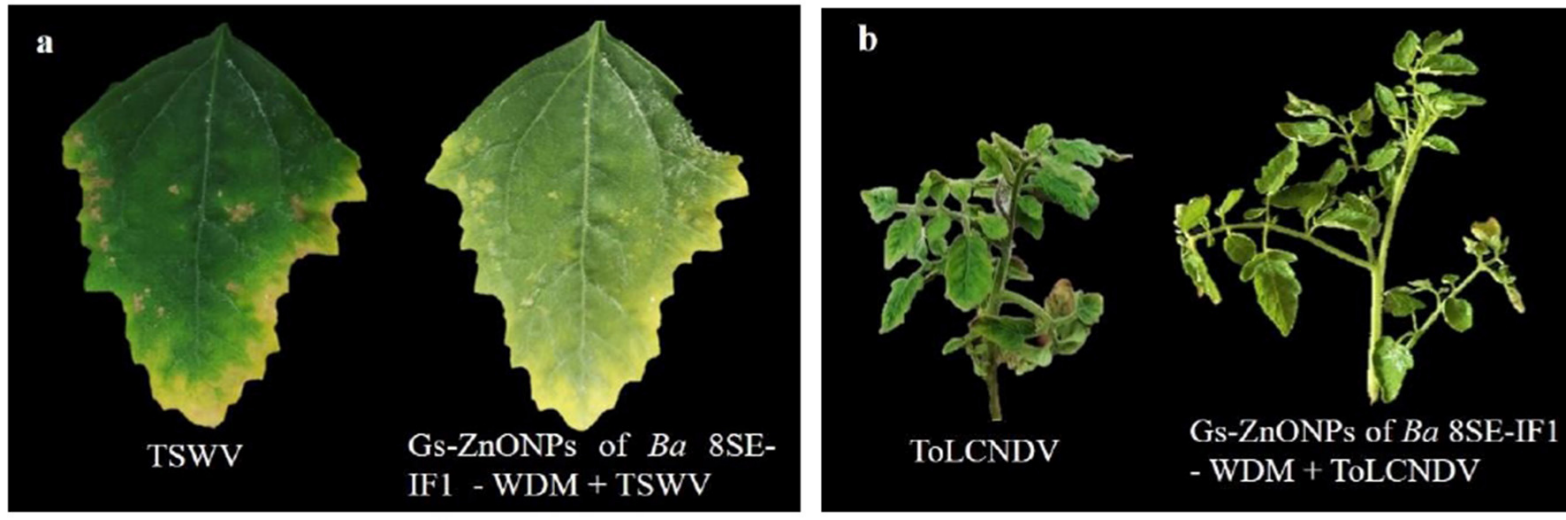
Effect of green synthesized ZnO nanoparticles of WDM of *Ba* 8SE-IF1 against viruses infecting tomato plants **(a)** TSWV - Tomato spotted wilt virus and **(b)** ToLCNDV - Tomato leaf curl New Delhi virus. Representative pictures from three independent experiments.

The antimicrobial properties of Gs-ZnO-NPs of *Ba*-8SE-IF1-WDM were again evaluated at 100 ppm against *P. aphanidermatum, P. nicotianae, F. oxysporum* f. sp. *lycopersici*, and *C. gloeosporioides* by the poisoned food technique; *R. solanacearum* and *X. campestris* by the agar well method; and TSWV and ToLCNDV by sap and graft transmission. Gs-ZnO-NPs of *Ba*-8SE-IF1-WDM significantly inhibited the growth of phytopathogenic oomycetes, fungi, and bacteria, and also the symptoms produced by TSWV and ToLCNDV (Table 3). The highest antifungal activity was observed against *F. oxysporum* f. sp. *lycopersici* with an inhibition of 84.6% (Table 3A; Figure 6c), followed by *C. gloeosporioides* (81.7%; Figure 6d), *P. nicotianae* (62.7%; Figure 6b), and *P. aphanidermatum* (43.1%; Figure 6a). In terms of antibacterial activity, the Gs-ZnO-NPs of *Ba*-8SE-IF1-WDM exhibited significant growth reduction of *R. solanacearum* with an inhibition zone of 21.24 mm (Table 3B; Figure 7a), followed by *X. campestris* (18.92 mm; Figure 7b). Furthermore, the antiviral potential of Gs-ZnO-NPs of *Ba*-8SE-IF1-WDM was evident, with 69.9% inhibition of local lesions produced by TSWV on *C. amaranticolor* (Table 3C; Figure 8a) and 62.6% inhibition of the vulnerability index of tomato leaf curl disease caused by ToLCNDV (Table 3D; Figure 8b). Thus, similar to the WDE of *Ba*-8SE-IF1, Gs-ZnO-NPs of *Ba*-8SE-IF1-WDM also exhibited simultaneous antifungal, antibacterial, and antiviral properties against different phytopathogens in tomato plants.

**Table 3:** Effect of green-synthesized zinc oxide nanoparticles of water-diffusible metabolites of *B. amyloliquefaciens* 8SE-IF1 against different phytopathogenic oomycetes, fungi, bacteria, and viruses infecting tomato plants.

**A**	**Radial mycelial growth (cm)**	**Percent inhibition**
Gs-ZnO-NPs of *Ba*-8SE-IF1-WDM *+ P. aphanidermatum*	5.12 ± 0.12^b^	43.1
Gs-ZnO-NPs of *Ba*-8SE-IF1-WDM *+ P. nicotianae*	3.35 ± 0.11^c^	62.7
Gs-ZnO-NPs of *Ba*-8SE-IF1-WDM *+ F. oxysporum f. sp. lycopersici*	1.38 ± 0.12^e^	84.6
Gs-ZnO-NPs of *Ba*-8SE-IF1-WDM *+ C. gloeosporioides*	1.64 ± 0.09^d^	81.7
Control	9.00 ± 0.00^a^	-
SE (m)	0.058	-
CD (0.05)	2.476	-
**B**	**Inhibition zone (mm)**	**-**
Gs-ZnO-NPs of *Ba*-8SE-IF1-WDM *+ R. solanacearum*	21.24 ± 0.07^a^	-
Gs-ZnO-NPs of *Ba*-8SE-IF1-WDM *+ X. campestris*	18.92 ± 0.25^b^	-
Control	0.0 ± 0.0^c^	-
SE (m)	0.115	-
CD (0.05)	1.277	-
**C**	**Number of lesions**	**Percent inhibition**
Gs-ZnO-NPs of *Ba*-8SE-IF1-WDM + TSWV	4.28 ± 0.54^b^	69.9
Control	14.23 ± 0.78^a^	-
SE (m)	0.278	-
CD (0.05)	0.968	-
**D**	**Vulnerability index**	**Percent inhibition**
Gs-ZnO-NPs of *Ba*-8SE-IF1-WDM+ ToLCNDV	26.60 ± 0.27^b^	62.6
Control	71.21 ± 0.84^a^	-
SE (m)	0.542	-
CD (0.05)	0.875	-

A: Oomycetes and fungal pathogens; B: Phytopathogenic bacteria; C: TSWV: tomato spotted wilt virus and D: ToLCNDV: tomato leaf curl New Delhi virus; Values are the mean of five replications ± standard deviation; SE, Standard error; CD, Critical difference (0.05); Superscripts with the same alphabets indicate on-par values, and those in different alphabets indicate a significant difference at the 5% level of significance.

### 3.6 Green-synthesized ZnO-NPs of WDM from *Ba*-8SE-IF1 promoted the growth and biomass of tomato plants

Tomato seedlings treated with Gs-ZnO-NPs alone at 100 ppm and the absolute control exhibited no incidence of bacterial wilt after 10 days of treatment. In contrast, plants inoculated with *R. solanacearum* alone showed symptoms, such as swelling of infected stems, green wilt, and eventually complete plant death, with a disease incidence of 60.3%. Surprisingly, Gs-ZnO-NPs of *Ba*-8SE-IF1-WDM at 100 ppm drastically reduced the incidence of bacterial wilt caused by *R. solanacearum* to a mere 9.9% (Table 4). The above data indicate the potential of using Gs-ZnO-NPs for the management of bacterial diseases in crop plants.

**Table 4:** Effect of green-synthesized ZnO nanoparticles of *Ba*-8SE-IF1-WDM against *R. solanacearum* inciting bacterial wilt and growth parameters of tomato plants.

**Treatments**	Percent disease incidence at 10 DAT^*^	Biometric observations at 20 DAT^*^					
		**Plant height (cm)**	**Number of branches/plants**	**Number of leaves/plant**	**Leaf area (cm** ^2)^	**Shoot biomass (g/plant)**	**Root biomass (g/plant)**
*R. solanacearum* alone at 10^8^ CFU/mL	60.3 ± 3.20^a^	10.55 ± 0.88^d^	1.20 ± 0.09^d^	10.35 ± 0.70^d^	1.25 ± 0. 27^d^	5.24 ± 0.25^d^	2.55 ± 0.14^d^
Gs-ZnO-NPs of *Ba*-8SE-IF1- WDM alone at 100 ppm	0.0 ± 0.0^c^	25.16 ± 0.32^a^	6.51 ± 0.28^a^	20.47 ± 0.82^a^	3.29 ± 0.14^a^	18.12 ± 1.28^a^	8.65 ± 1.54^c^
Gs-ZnO-NPs of *Ba*-8SE-IF1-WDM at 100 ppm + *R. solanacearum* at 10^8^ CFU/mL	9.9 ± 0.85^b^	20.47 ± 0.58^b^	4.29 ± 0.19^b^	18.20 ± 0.54^b^	2.62 ± 0.04^b^	12.65 ± 1.07^b^	6.48 ± 1.02^c^
Absolute control	0.0 ± 0.0^c^	15.01 ± 0.14^c^	2.95 ± 0.06^c^	13.25 ± 0.83^c^	1.61 ± 0.15^c^	7.92 ± 0.94^c^	3.24 ± 0.54^c^
SE (m)	0.599	0.422	0.135	0.567	0.195	0.917	0.681
CD (0.05)	2.038	1.311	1.116	1.279	1.010	1.278	1.042

* DAT, Days after treatment, values are the mean of 10 replications each from three independent experiments ± standard deviation; SE, Standard error; CD, Critical difference (0.05). Superscripts with the same alphabet indicate on-par values, and those with different alphabets indicate a significant difference at the 5% level of significance.

**Figure 9:**
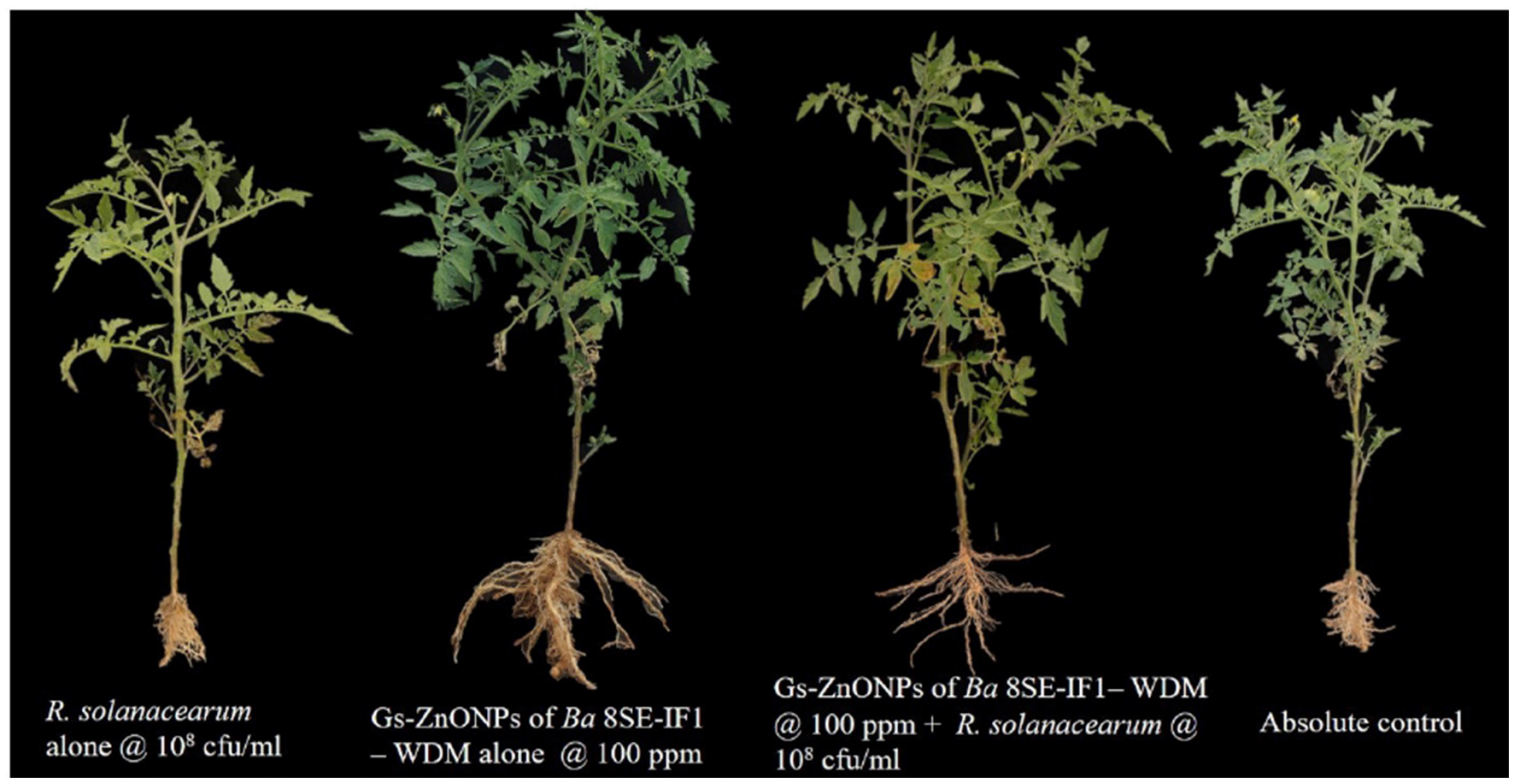
Effect of green synthesized ZnO nanoparticles of *Ba*-8SE-IF1 - WDM on growth parameters of tomato plants. Representative pictures from three independent experiments.

The biometric parameters, including plant height (cm), number of branches per plant, number of leaves per plant, leaf area (cm^2^), and shoot and root biomass (g/plant), were assessed 20 days after the treatments. The maximum plant height was observed in plants treated with Gs-ZnO-NPs alone at 100 ppm (25.16 ± 0.32 cm). This was followed by combined treatment of the Gs-ZnO-NPs and *R. solanacearum* inoculation, which recorded a plant height of 20.47 ± 0.58 cm. In contrast, the absolute control recorded the plant height of 15.01 ± 0.14 cm, whereas *R. solanacearum* alone treated plants showed death of more than 60% of plants, and the surviving plants had the least plant height of 10.55 ± 0.88 cm (Table 4; Figure 9). Similarly, the maximum number of branches per plant was observed in the Gs-ZnO-NPs-treated plants (6.51 ± 0.28 branches/plant), followed by the Gs-ZnO-NPs and *R. solanacearum-*treated plants (4.29 ± 0.19 branches/plant). The absolute control had 2.95 ± 0.06 branches/plant, and the survived bacterial pathogen-inoculated plants had the significantly lowest number of branches (1.20 ± 0.09 branches/plant; Table 4; Figure 9).

A similar trend was also observed in the number of leaves and the leaf area. The maximum number of leaves per plant and leaf area were recorded in Gs-ZnO-NP-treated plants compared with Gs-ZnO-NPs and *R. solanacearum*. The control plants had a comparatively smaller number of leaves and leaf area, whereas the surviving plants of *R. solanacearum* inoculation showed significantly lower leaf counts and leaf area (Table 4; Figure 9). The Gs-ZnO-NPs had a tremendous effect on shoot and root biomass, measured as fresh weight (g/plant), at the maximum vegetative growth of tomato plants. The highest shoot and root biomass was recorded in plants treated with the Gs-ZnO-NPs (18.12 ± 1.28; 8.65 ± 1.54 g/plant), followed by the Gs-ZnO-NPs and *R. solanacearum* infection (12.65 ± 1.07; 6.48 ± 1.02 g/plant). In comparison, the absolute control (7.92 ± 0.94; 3.24 ± 0.54 g/plant) and the survived *R. solanacearum*-infected plants recorded significantly lower shoot and root biomass (5.24 ± 0.25; 2.55 ± 0.14; Table 4; Figure 9). The above data clearly indicate the growth promotion potential of Gs-ZnO-NPs of *Ba*-8SE-IF1-WDM in crop plants.

## 4 Discussion

The present study focused on the extraction and identification of antimicrobial compounds from WDE of a promising endophytic and antagonistic bacterium, *B. amyloliquefaciens* 8SE-IF1; green synthesis of zinc oxide nanoparticles using WDM of *Ba*-8SE-IF1; and evaluation of antimicrobial activity of *Ba*-8SE-IF1-WDE and its Gs-ZnO-NPs against different phytopathogenic oomycetes, fungi, bacteria, and viruses infecting tomato plants. WDE of *Ba*-8SE-IF1 exhibited significant broad-spectrum antimicrobial activities against *P. aphanidermatum, P. nicotianae, F. oxysporum* f. sp. *lycopersici, C. gloeosporioides, R. solanacearum*, and *X. campestris* and also resulted in remission of symptoms caused by TSWV and ToLCNDV. The inhibition of these phytopathogens indicates that the WDE of *Ba*-8SE-IF1 contains multiple antimicrobial compounds. GC-MS/MS analysis revealed that WDE contains at least 26 major organic compounds and has antifungal, antibacterial, antiviral, or simultaneous antimicrobial properties. The identified antimicrobial compounds have functional groups such *as* phenols, carboxylic acids, alcohols, carbonyl groups of aldehydes, and aliphatic hydrocarbons. Moreover, five compounds, *viz.*, phenol 3,5-bis (1,1-dimethyl-ethyl), hexadecane, 1-tetradecene, 2,6,10,14-tetramethyl hexadecane, 2,6,11, and 15-tetramethyl hexadecane, have antifungal, antibacterial, antiviral, and antimicrobial properties. These abilities enabled endophytic bacteria to significantly inhibit the growth of phytopathogenic oomycetes, fungi, and bacteria, as well as reduce the symptoms produced by TSWV and ToLCNDV. This is the premier study that reports simultaneous inhibition of a multitude of phytopathogens belonging to different kingdoms by WDE of an endophytic and antagonistic bacterium in crop plants. However, the antimicrobial properties of culture filtrates of antagonistic bacteria have been reported against either phytopathogenic fungi or bacteria. A 40% culture filtrate of *Bacillus* sp. B44 inhibited the growth of *F. oxysporum* f. sp. *lycopersici* to 70% (Jangir et al., 2018), a 50% culture filtrate of *B. licheniformis* suppressed the growth of *R. solani, C. gloeosporioides*, and *P. capsici* (Jeong et al., 2017), and the culture filtrate of *Pseudomonas aeruginosa* Os_12 impeded *F. oxysporum* f. sp. *pisi* (Gupta et al., 2022). The culture filtrates of *Pseudomonas kilonensis* Ba35 and *Serratia liquefaciens* Ou55 inhibited *Agrobacterium tumifaciens* (Etminani et al., 2024), and volatile compounds from *Bacillus* strain D13 reduced the growth of *X. oryzae* pv. *oryzae* (Xie et al., 2018). The fatty acids from *B. amyloliquefaciens* VB7 had antiviral activity against the tobacco streak virus in cotton (Vinodkumar et al., 2018).

GC-MS/MS analysis also revealed the presence of major functional groups, including phenols, carboxylic acids, alcohols, aldehydes (carbonyl compounds), and aliphatic hydrocarbons (alkanes) in the Gs-ZnO-NPs of WDM of the promising endophytic bacterial strain. Phenols and carboxylic acid compounds are known to possess antifungal properties by directly interacting with fungal cell membranes. These compounds integrate into the lipid bilayer, increasing membrane fluidity and leading to disorganization and eventual cell lysis (Avis and Bélanger, 2001). The phenolic compound, phenol 2,4-bis (1,1-dimethylethyl), exhibited antifungal activity against *Alternaria solani* and *Botrytis cinerea* by disrupting membranes, inhibiting lipid peroxidation, and inducing cell death (Gao et al., 2017). Hexadecanoic acid is fungistatic and targets the fungal cell wall and interferes with ergosterol biosynthesis in *F. oxysporum, A. solani*, and *C. lagenarium* (Liu et al., 2008). The aliphatic hydrocarbons primarily exhibit antifungal activity by inhibiting spore germination (Prakash and Arora, 2021) and causing mycelial disruption and distortion (Muhialdin et al., 2020). Similarly, alcohols and aldehydes exhibit antibacterial activity by inducing potassium ion (K^+^) leakage from bacterial cells, resulting in membrane disruption and structural damage (Togashi et al., 2007). Oleic and hexadecanoic acids disrupt the membranes of *Pseudomonas syringae, R. solanacearum*, and *X. campestris*, resulting in increased permeability, oxidative stress, and metabolic disruption (Sohn et al., 2013; Idris, 2022), and interrupt the entry and movement of tobacco mosaic virus by altering the lipid makeup of the host cell membrane (Zhao et al., 2017). Pentadecenoic, heptadecenoic, and octadecenoic acids produced by *B. amyloliquefaciens* VB7 synergistically suppress tobacco streak virus replication in cotton (Vinodkumar et al., 2018). However, the antiviral mechanisms of these compounds remain unclear.

The green synthesis of ZnO nanoparticles was confirmed by the immediate color change of the reaction mixtures after the incubation period. A yellowish-brown color was observed, while green-synthesizing nanoparticles of *Ba*-8SE-IF1-WDM were observed. Similarly, white color changes were observed in the green synthesis of zinc nanoparticles of *Pseudomonas fluorescens* (Vinay et al., 2018). The color changes observed in aqueous solutions are attributed to the plasmon resonance phenomenon. This process is involved in the reduction of zinc metallic salts into nanoparticles and is facilitated by the oxidation of aldehyde groups in biomolecules into carboxylic acids (Shameli et al., 2012). The peak of UV-Vis absorption observed at 387 nm for the Gs-ZnO-NPs of *Ba*-8SE-IF1-WDM confirms the formation of biologically active monodisperse ZnO-NPs in the solution. The observed absorption peak is due to the surface plasmon resonance directly linked to the particular size, shape, and composition of the solution used in the nanoparticle synthesis. Comparable absorption spectra were observed for green-synthesized zinc oxide nanoparticles of *Cinnamomum camphora* leaf extracts at 368–374 nm (Zhu et al., 2021) and *Bacillus cereus* RNT6 at 382 nm (Ahmed et al., 2021).

The stability of the Gs-ZnO-NPs of *Ba*-8SE-IF1-WDM was assessed based on its zeta potential. It resulted in −33.1 mV, which demonstrated the stability of the antimicrobial metabolites in the Gs-NPs. A high magnitude of either positive or negative zeta potential indicates strong electrostatic repulsion between the Gs-NPs, which prevents their aggregation and ensures long-term stability. Moreover, this property ensures the consistent biological activity of the Gs-NPs in inhibiting phytopathogens and the symptoms produced by them, in addition to growth promotion in crop plants. Singh et al. (2019) reported a zeta potential of −17.1 mV for ZnO-NPs of the leaf extract of *Punica granatum* L.

The FTIR spectrum of the GS-ZnO-NPs confirms the presence of O-H stretching in alcohols and phenolic compounds, in addition to carbonyl (C=O) groups in the antimicrobial compounds present in the water-diffusible metabolites of *Ba*-8SE-IF1. Similarly, the FTIR spectrum shows the presence of O-H and N-H stretching of aliphatic primary amines, broad O-H stretching, and amide I b and C-N stretching of amines in the biologically active Gs-ZnO-NPs. These properties of other Gs-NPs were reported by Fouda et al. (2018, 2020), Mohamed et al. (2019), and Mohamed et al. (2021). X-ray diffraction of Gs-ZnO-NPs of *Ba*-8SE-IF1-WDM reinstated its crystalline structure, whereas the sharp peak confirmed its purity and crystallinity.

FE-SEM analysis revealed that the Gs-ZnO-NPs exhibited an oval to spherical shape with a size of 59.94 nm. The spherical form of nanoparticles indicates the minimization of the total surface energy, isotropic growth conditions (even distribution of reducing agents), and uniform encapsulation of biomolecules, which in turn enhances their biological properties and performance. Comparable results have been reported in the shape of other green-synthesized ZnO nanoparticles, i.e., spherical particles of 21–35 nm (Ahmed et al., 2021), irregular particles of ~100 nm (Mohamed et al., 2019), and hexagonal particles with an average size of 50–90 nm (Gupta et al., 2018).

The Gs-ZnO-NPs exhibited substantially high antimicrobial activity against all the tested phytopathogens, underscoring their role in enhancing different biological functions. These Gs-NPs interact electrostatically with microbial membranes to facilitate their cellular uptake. They generate redox-active electrons to produce reactive oxygen species (ROS), which in turn damage key cellular components such as proteins, lipids, enzymes, and nucleic acids of phytopathogenic microbes (Sánchez-López et al., 2020). Additionally, NPs interfere with protein function, disrupt membrane integrity, alter cell morphology, and induce cytoplasmic leakage (Díez-Pascual, 2018; Kalia et al., 2020). The Gs-ZnO-NPs have a lower risk of resistance development due to their multiple mechanisms of action against plant pathogens, as they contain at least 26 major antimicrobial compounds. This contrasts with commercial crop protection chemicals, which typically have a single mode of action, making it easier for pathogens to adapt and develop resistance over time. There are very limited reports on the antimicrobial activity of Gs-ZnO-NPs from beneficial microbes against multiple phytopathogens. However, plant-based Gs-ZnO-NPs were developed and reported to inhibit plant pathogens, *viz., Alternaria mali, Botryosphaeria dothidea*, and *Diplodia seriata* (Ahmad et al., 2020); *Alternaria alternata* (Zhu et al., 2021); *Aspergillus niger* (Kumawat et al., 2025); *X. oryzae* pv. *oryzae* (Ogunyemi et al., 2019); *R. solanacearum, Erwinia carotovora*, and *Clavibacter michiganensis* (Rashid et al., 2024); and tobacco mosaic virus (Abdelkhalek and Al-Askar, 2020).

Tomato seedlings inoculated with *R. solanacearum* showed a high bacterial wilt incidence of 60.3%, whereas Gs-ZnO-NPs of *Ba*-8SE-IF1-WDM-treated plants showed an enhanced tolerance to *R. solanacearum*, resulting in a lower incidence of 9.9%. The observed reduction in disease incidence is attributed to the antibacterial properties of the Gs-ZnO-NPs of *Ba*-8SE-IF1-WDM. 1-hexadecanol, 4,6-dimethyl dodecane, 5-methyl tetradecane, 2,6,10,15-tetramethyl heptadecane, heneicosane, and n-tetradecane present in Gs-NPs are reported to inhibit bacterial proliferation by disrupting tolerance mechanisms, impairing transmembrane proton translocation, and suppressing glycolytic activity (Applerot et al., 2009; Reddy et al., 2007). Interestingly, Gs-ZnO-NPs did not show any inhibitory effects on commonly used biocontrol agents such as *P. fluorescens* and *Trichoderma* spp. (data not shown).

The commercial fungicides (metalaxyl for oomycetes and carbendazim for fungi at 100 ppm) exhibited antifungal activity against *F. oxysporum* f. sp. *lycopersici* (47.7%), followed by *C. gloeosporioides* (36.6%), *P. nicotianae* (32.2%), and *P. aphanidermatum* (19.44%) in the poisoned food assay. Streptocycline (100 ppm) showed antibacterial activity against *R. solanacearum* and *X. campestris*, with inhibition zones of 13.25 mm and 11.47 mm, respectively, in the agar well method. Under *in vivo* conditions, streptocycline (100 ppm)-treated plants showed green wilt symptoms, with a disease incidence of 20.8%. However, WDE and Gs-NPs outperformed the commercial fungicides and antibiotics used for the management of fungal and bacterial diseases in tomatoes.

Furthermore, the biometric parameters were significantly improved in tomato plants treated with Gs-ZnO-NPs of *Ba*-8SE-IF1-WDM. There was a 35 to 100% increase in the different biometric parameters of vegetative growth compared to the control plants. It also showed at least a 25–90% improvement in the vegetative parameters compared to either metalaxyl- or carbendazim-treated plants (data not shown). Zinc plays a crucial role as a micronutrient in plant growth and development, functioning as a cofactor for numerous enzymes involved in protein synthesis, auxin metabolism, and cell division. However, zinc applied through conventional fertilizers is often immobilized in the soil, limiting its availability to plants (Sharma et al., 2022). The nanoscale formulation of the Gs-ZnO-NPs of WDM enhances zinc solubility and bioavailability, thus enabling its efficient uptake and translocation within plant tissues. This improved zinc nutrition is probably responsible for the observed stimulation of vegetative growth.

To summarize, this study clearly demonstrates that WDE of *Ba*-8SE-IF1 and its Gs-ZnO-NPs exhibited synchronized antioomycete, antifungal, antibacterial, and antiviral activities against an array of phytopathogens in addition to growth-promoting effects in tomato plants. WDE and its Gs-ZnO-NPs have at least 26 major compounds that are responsible for their antimicrobial properties. Thus, they are highly promising candidates for the development of nanopesticide formulations. The experimental results should be validated under multi-location field trials in different agroecological units. Additional studies are needed to understand the complex interactions between green-synthesized nanoparticles and tomato plants under diverse agroecological conditions. In addition, the mechanism underlying the plant growth-promoting potential of green-synthesized nanoparticles remains to be elucidated. Such investigations are very crucial in assessing the long-term efficacy of Gs-NPs to facilitate the development of sustainable nanotechnology in crop production and protection systems.

## Data Availability

The original contributions presented in the study are publicly available. This data can be found in in the NCBI GenBank (https://www.ncbi.nlm.nih.gov/genbank/). The Ralstonia solanacearum sequence is available under the accession number PV022497 and the Bacillus amyloliquefaciens sequence is available under the accession number PV023912.
