# Supplementary material for: Endophytic and antagonistic Bacillus amyloliquefaciens 8SE-IF1-derived nanoparticles encumber phytopathogenic oomycetes, fungi, bacteria, and viruses with enhanced growth in tomato seedlings
Source: Front Microbiol. 2025 Aug 1;16:1612335. doi: 10.3389/fmicb.2025.1612335 (PMC12354652; doi:10.3389/fmicb.2025.1612335)
Supplement: Supplementary file 1 [file Data_Sheet_1.pdf]

Endophytic and antagonistic *Bacillus amyloliquefaciens* 8SE-IF1-derived nanoparticles encumber phytopathogenic oomycetes, fungi, bacteria, and viruses with enhanced growth in tomato seedlings

Mary Sharmila A.[1*], Joy Michal Johnson[1*], Saru Sara Sam[1], Deepa R. Chandran[1], Ajay B.[1], Heera G.[1], Sarada S.[2], Usha C. Thomas[3,] Swapna Alex[4] and Radhakrishnan N.V.[1]

Supplementary data:

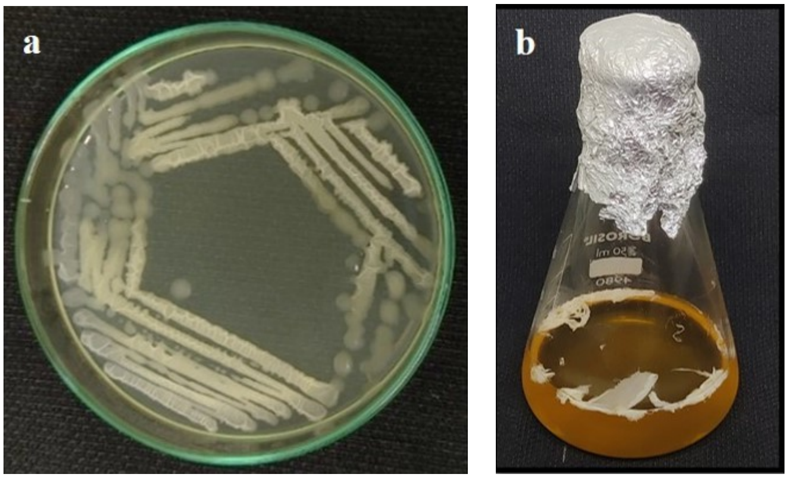

Supplementary Fig. S1

Pure culture of most promising endophytic and antagonistic bacterial strain *Bacillus amyloliquefaciens* 8SE-IF1 on a) nutrient agar medium; b) in nutrient broth

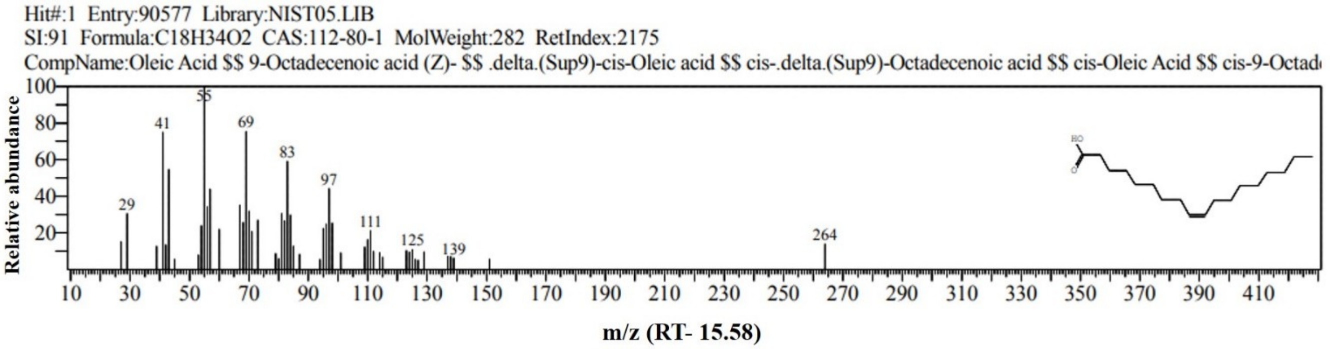
Hit#:1 Entry:90577 Library:NIST05.LIB
SI:91 Formula:C18H34O2 CAS:112-80-1 MolWeight:282 RetIndex:2175
CompName:Oleic Acid $$ 9-Octadecenoic acid (Z)- $$ .delta.(Sup9)-cis-Oleic acid $$ cis-.delta.(Sup9)-Octadecenoic acid $$ cis-Oleic Acid $$ cis-9-Octad
Relative abundance
29
41
55
69
83
97
111
125
139
264
m/z (RT- 15.58)

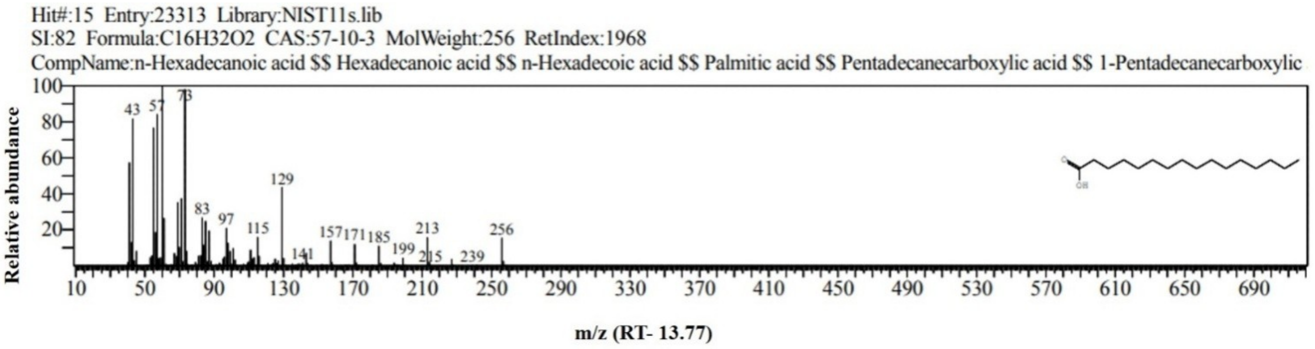
Hit#:15 Entry:23313 Library:NIST11s.lib
SI:82 Formula:C16H32O2 CAS:57-10-3 MolWeight:256 RetIndex:1968
CompName:n-Hexadecanoic acid $$ Hexadecanoic acid $$ n-Hexadecoic acid $$ Palmitic acid $$ Pentadecanecarboxylic acid $$ 1-Pentadecanecarboxylic
Relative abundance
43
57
73
83
97
115
129
141
157
171
185
199
213
215
239
256
m/z (RT- 13.77)

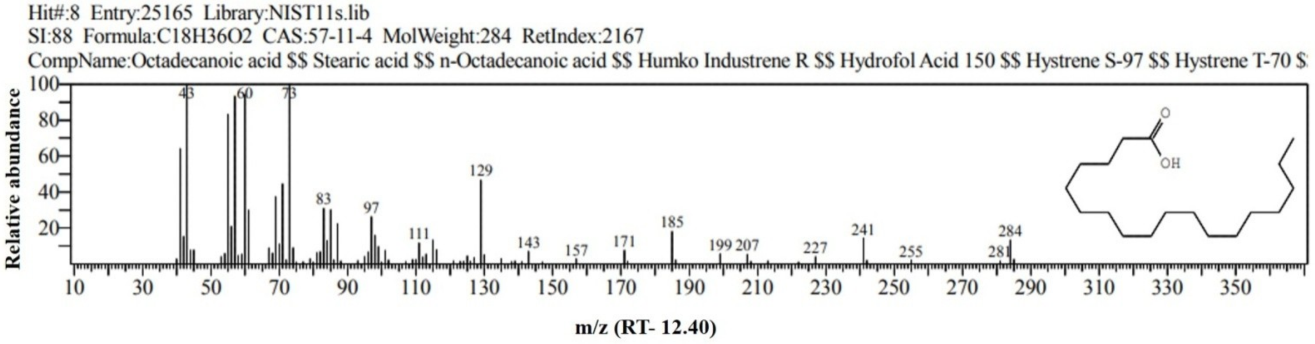
Hit#:8 Entry:25165 Library:NIST11s.lib
SI:88 Formula:C18H36O2 CAS:57-11-4 MolWeight:284 RetIndex:2167
CompName:Octadecanoic acid $$ Stearic acid $$ n-Octadecanoic acid $$ Humko Industrene R $$ Hydrofol Acid 150 $$ Hystrene S-97 $$ Hystrene T-70 $
Relative abundance
43
60
73
83
97
111
129
143
157
171
185
199
207
227
241
255
281
284
OH
m/z (RT- 12.40)

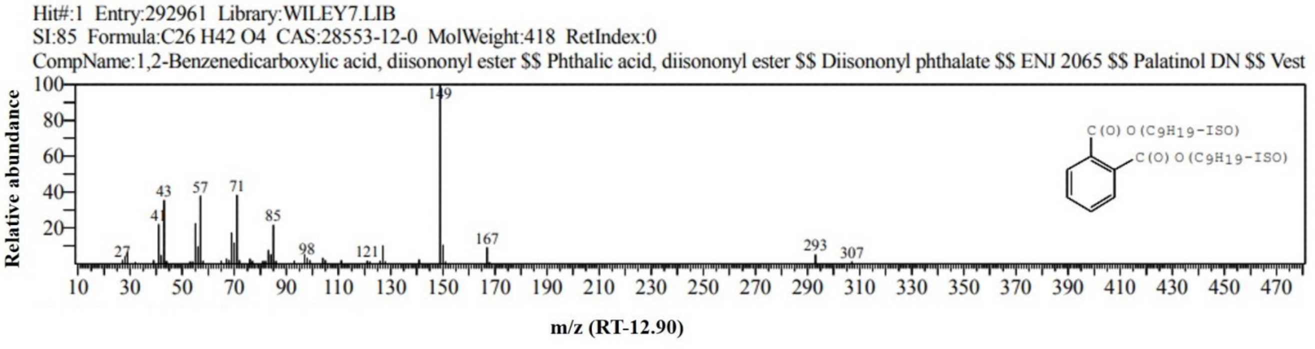
Hit#:1 Entry:292961 Library:WILEY7.LIB
SI:85 Formula:C26 H42 O4 CAS:28553-12-0 MolWeight:418 RetIndex:0
CompName:1,2-Benzenedicarboxylic acid, diisononyl ester $$ Phthalic acid, diisononyl ester $$ Diisononyl phthalate $$ ENJ 2065 $$ Palatinol DN $$ Vest
Relative abundance
27
41
43
57
71
85
98
121
149
167
293
307
C(O)O(C9H19-ISO)
C(O)O(C9H19-ISO)
m/z (RT-12.90)

Hit#:1 Entry:18383 Library:NIST11s.lib
SI:94 Formula:C14H22O CAS:1138-52-9 MolWeight:206 RetIndex:1555
CompName:Phenol, 3,5-bis(1,1-dimethylethyl)- $$ Phenol, 3,5-di-tert-butyl- $$ 3,5-Di-tert-butylphenol $$ Phenol, 3,5-bis(t-butyl) $$ 3,5-Di-t-butylphenol $

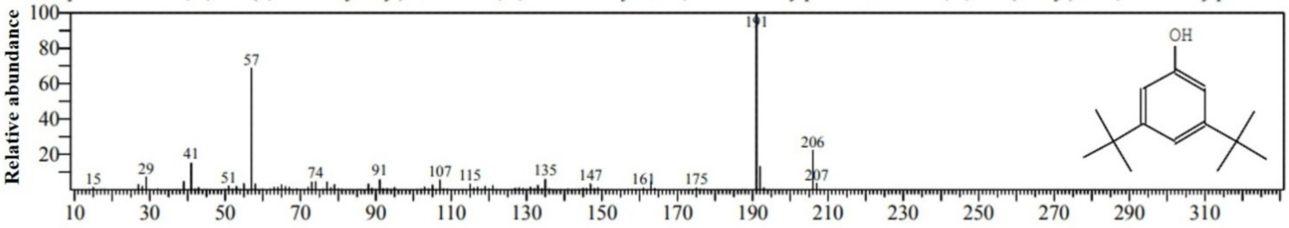

m/z (RT- 8.72)

Hit#:1 Entry:106265 Library:NIST11.lib
SI:97 Formula:C19H40O CAS:1454-84-8 MolWeight:284 RetIndex:2153
CompName:n-Nonadecanol-1 $$ 1-Nonadecanol $$ Nonadecanol $$ Nonadecan-1-ol $$ Nonadecyl alcohol $$

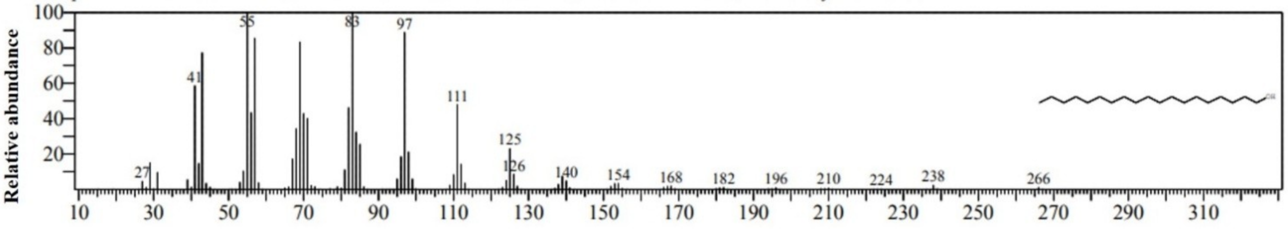

m/z (RT-11.86)

Hit#:9 Entry:283535 Library:WILEY7.LIB
SI:94 Formula:C21 H14 FE N2 O3 CAS:74764-11-7 MolWeight:398 RetIndex:0
CompName:Iron, tricarbonyl[N-(phenyl-2-pyridinylmethylene)benzenamine-N,N']- $$ IRON, TRICARBONYL[N-(PHENYL-2-PYRIDINYLMETHYLEN

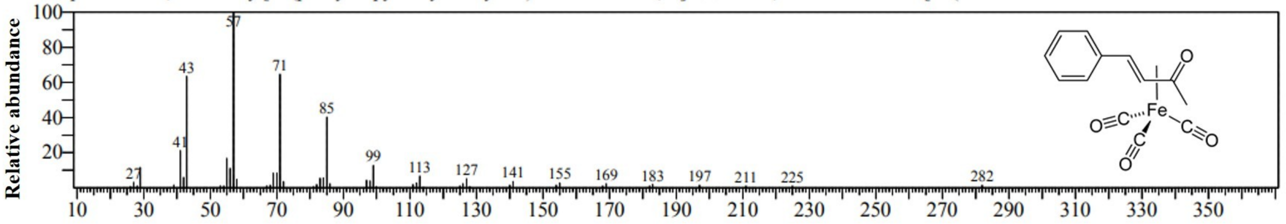

m/z (RT-11.43)

Hit#:1 Entry:14425 Library:NIST05s.LIB
SI:91 Formula:C12H26O CAS:3913-02-8 MolWeight:186 RetIndex:1393
CompName:1-Octanol, 2-butyl- $$ 2-Butyl-1-octanol $$ 2-Butyloctanol $$ 2-Butyloctyl alcohol $$ 5-(Hydroxymethyl)undecane $$ Isododecyl alcohol $$ M

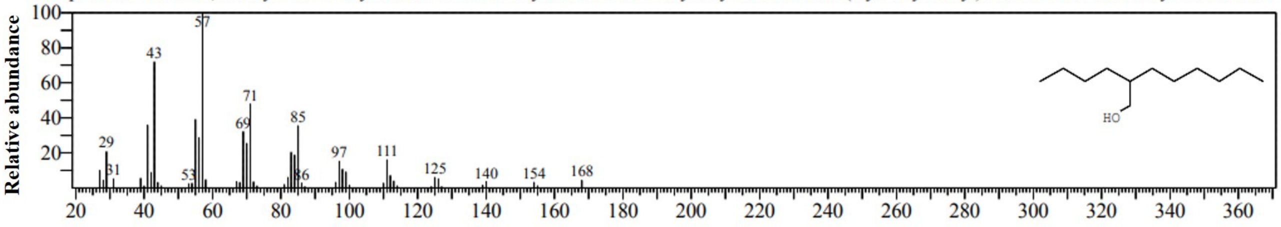

m/z (RT-10.77)

Hit#:15 Entry:20389 Library:NIST05s.LIB
SI:95 Formula:C16H34O CAS:36653-82-4 MolWeight:242 RetIndex:1854
CompName:1-Hexadecanol $$ n-Cetyl alcohol $$ n-Hexadecan-1-ol $$ n-Hexadecanol $$ n-1-Hexadecanol $$ Adol 52 $$ Adol 52 NF $$ Adol 54 $$ Aldo

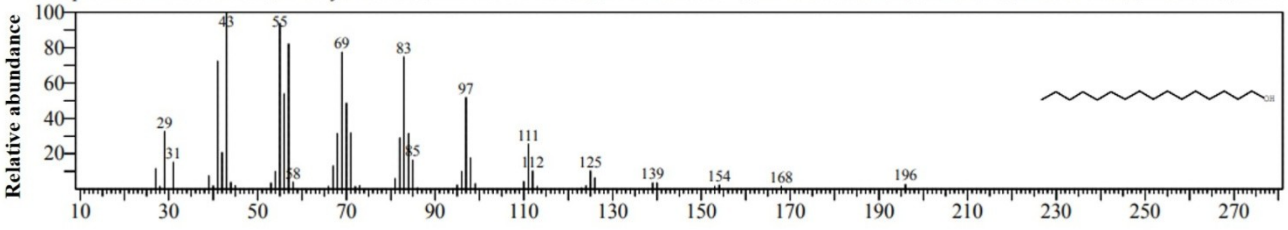

m/z (RT- 9.53)

Hit#:1 Entry:17325 Library:NIST11s.lib
SI:93 Formula:C14H30 CAS:61141-72-8 MolWeight:198 RetIndex:1285
CompName:Dodecane, 4,6-dimethyl- $$ 4,6-Dimethyldodecane # $$

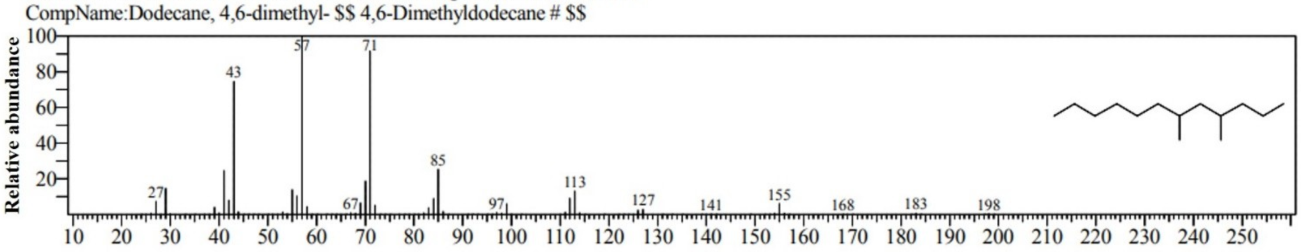

m/z (RT-5.31)

Hit#:1 Entry:48596 Library:NIST05.LIB
SI:94 Formula:C15H32 CAS:25117-32-2 MolWeight:212 RetIndex:1448
CompName:Tetradecane, 5-methyl- $$ 5-Methyltetradecane # $$

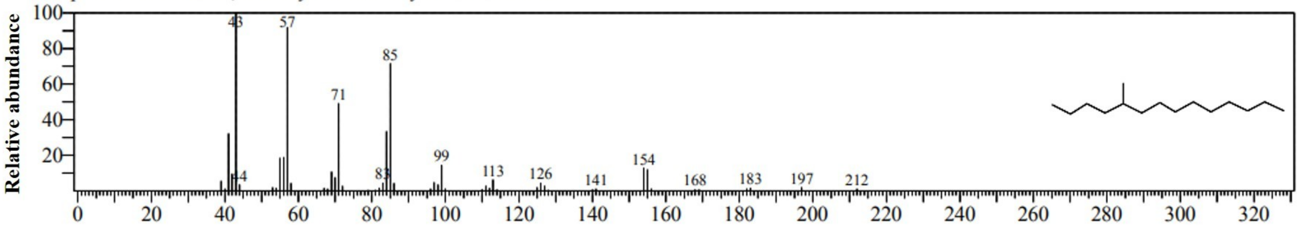

m/z (RT- 5.42)

Hit#:17 Entry:234107 Library:WILEY7.LIB
SI:91 Formula:C23 H48 CAS:638-67-5 MolWeight:324 RetIndex:0
CompName:Tricosane (CAS) n-Tricosane $$

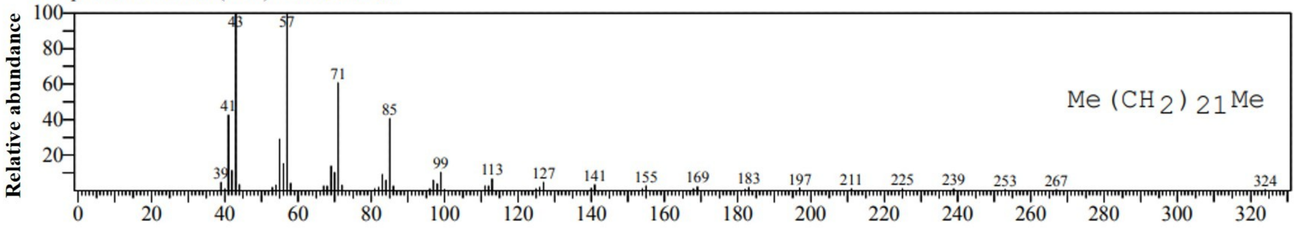

m/z (RT-5.62)

Hit#:12 Entry:32384 Library:NIST05.LIB
SI:90 Formula:C13H28 CAS:17301-30-3 MolWeight:184 RetIndex:1185
CompName:Undecane, 3,8-dimethyl- $$ 3,8-Dimethylundecane # $$

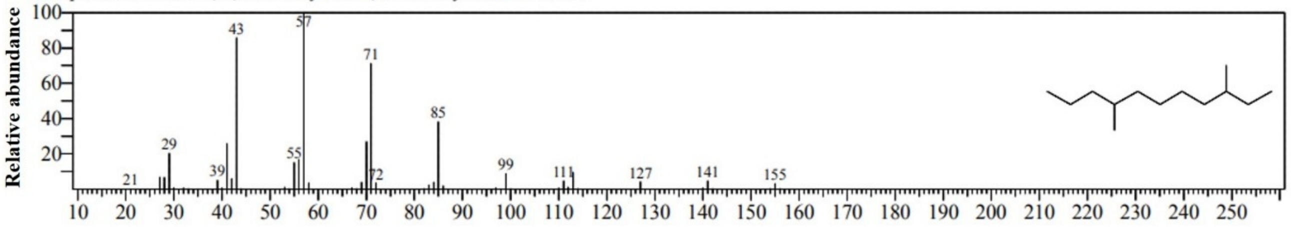

m/z (RT-5.90)

Hit#:13 Entry:57195 Library:NIST05.LIB
SI:90 Formula:C16H34 CAS:544-76-3 MolWeight:226 RetIndex:1612
CompName:Hexadecane $$ n-Cetane $$ n-Hexadecane $$ Cetane $$

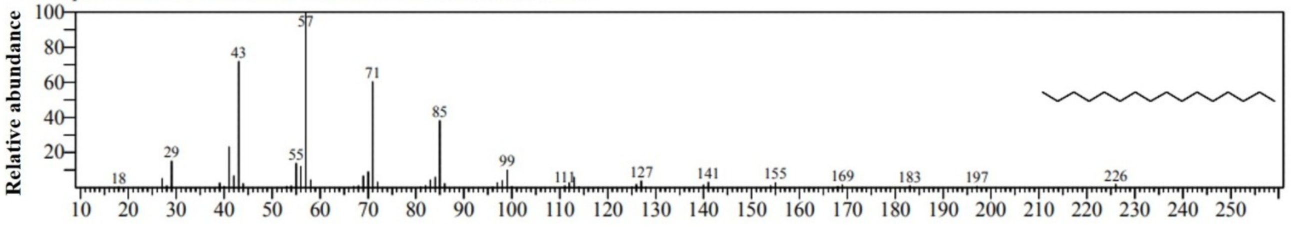

m/z (RT-6.19)

Hit#:1 Entry:39069 Library:NIST05.LIB
SI:97 Formula:C14H28 CAS:1120-36-1 MolWeight:196 RetIndex:1403
CompName:1-Tetradecene $$ n-Tetradec-1-ene $$ .alpha.-Tetradecene $$ Neodene 14 $$ Tetradec-1-ene $$ Tetradecene-1 $$

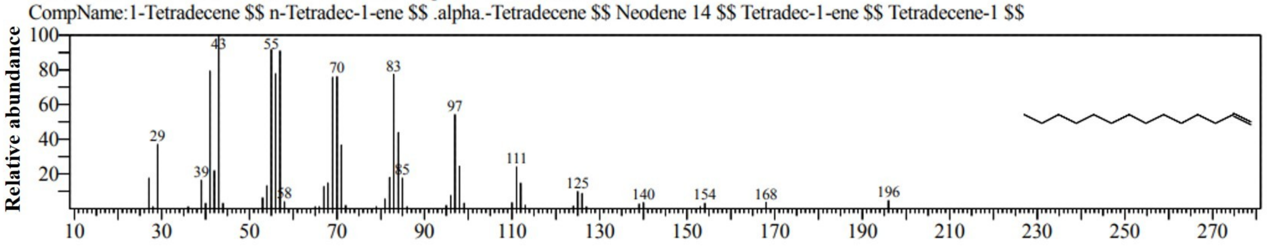

m/z (RT-7.03)

Hit#:11 Entry:104536 Library:NIST11.lib
SI:93 Formula:C20H42 CAS:13287-24-6 MolWeight:282 RetIndex:1945
CompName:Nonadecane, 9-methyl- $$ 9-Methylnonadecane # $$ 9-Methylnonadecane $$

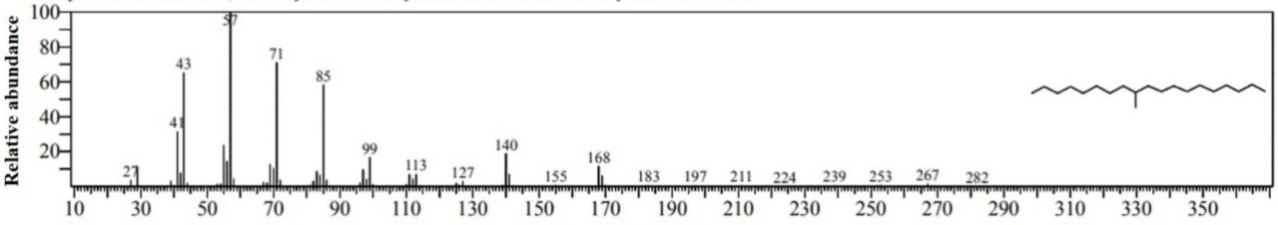

m/z (RT-7.40)

Hit#:1 Entry:193409 Library:WILEY7.LIB
SI:94 Formula:C20 H42 CAS:638-36-8 MolWeight:282 RetIndex:0
CompName:Hexadecane, 2,6,10,14-tetramethyl- (CAS) Phytane $$ 2,6,10,14-Tetramethylhexadecane $$ Phytan $$ HEXADECAN, 2,6,10,14-TETRAMET

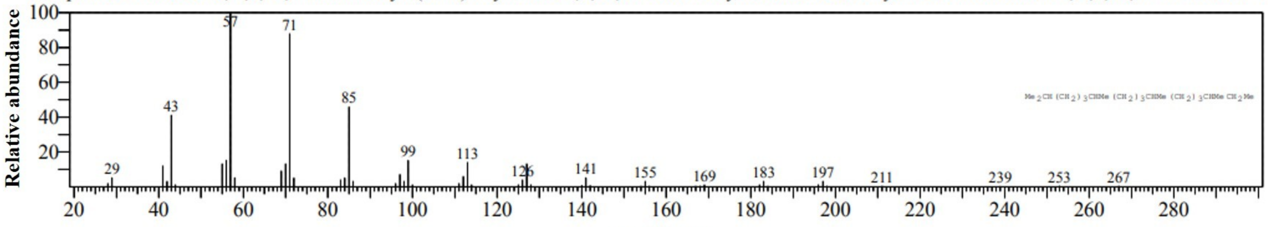

m/z (RT-7.90)

Hit#:16 Entry:98901 Library:NIST05.LIB
SI:92 Formula:C21H44 CAS:54833-48-6 MolWeight:296 RetIndex:1852
CompName:Heptadecane, 2,6,10,15-tetramethyl- $$ 2,6,10,15-Tetramethylheptadecane # $$

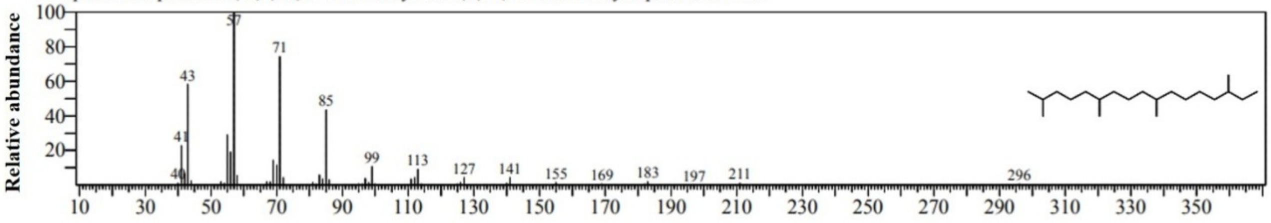

m/z (RT-8.26)

Hit#:16 Entry:29628 Library:NIST11s.lib
SI:91 Formula:C28H58 CAS:630-02-4 MolWeight:394 RetIndex:2804
CompName:Octacosane $$ n-Octacosane $$

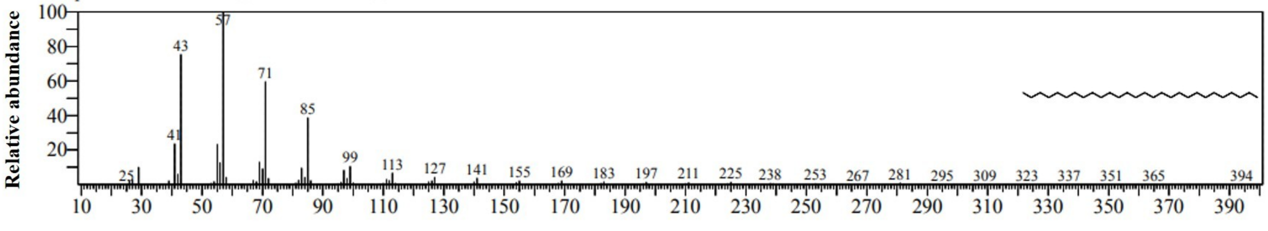

m/z (RT- 8.35)

Hit#:9 Entry:22094 Library:NIST05s.LIB
SI:92 Formula:C19H40 CAS:629-92-5 MolWeight:268 RetIndex:1910
CompName:Nonadecane $$ n-Nonadecane $$

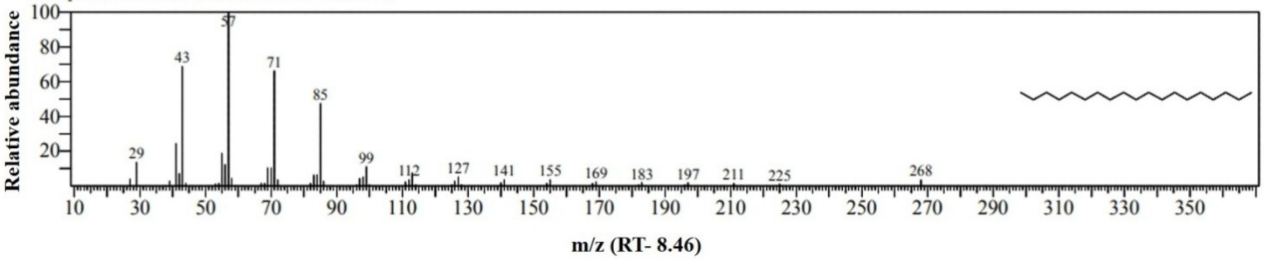

Hit#:2 Entry:20226 Library:NIST05s.LIB
SI:93 Formula:C17H36 CAS:629-78-7 MolWeight:240 RetIndex:1711
CompName:Heptadecane $$ n-Heptadecane $$ Normal-heptadecane $$

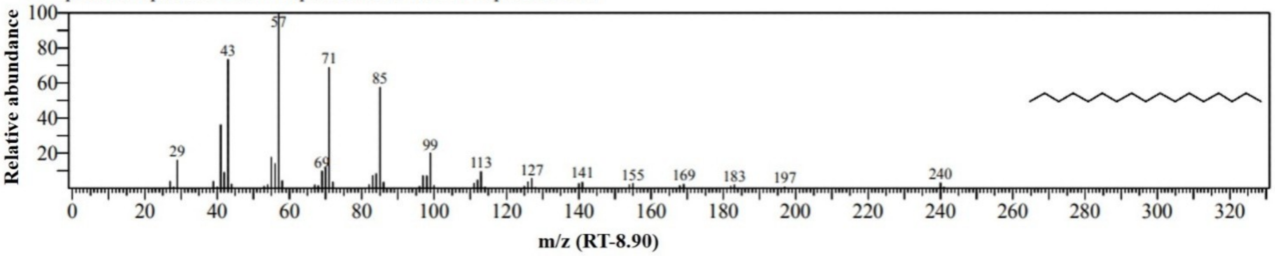

Hit#:15 Entry:90685 Library:NIST05.LIB
SI:91 Formula:C20H42 CAS:504-44-9 MolWeight:282 RetIndex:1753
CompName:Hexadecane, 2,6,11,15-tetramethyl- $$ Crocetane $$ 2,6,11,15-Tetramethylhexadecane $$

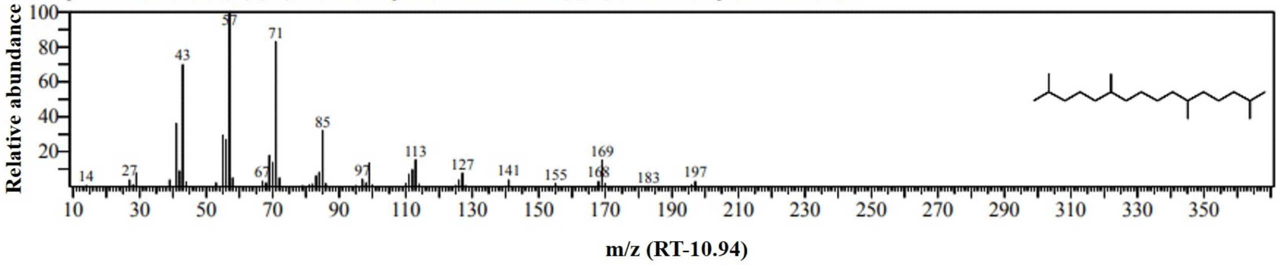

Hit#:12 Entry:185354 Library:NIST11.lib
SI:90 Formula:C29H60 CAS:0-00-0 MolWeight:408 RetIndex:2840
CompName:2-methyloctacosane

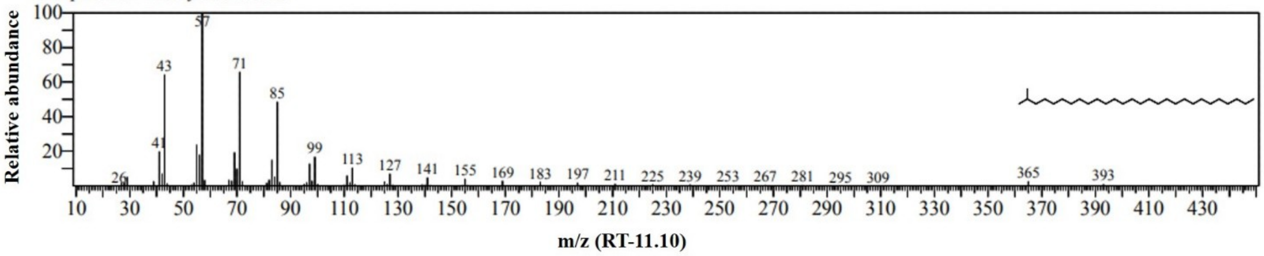

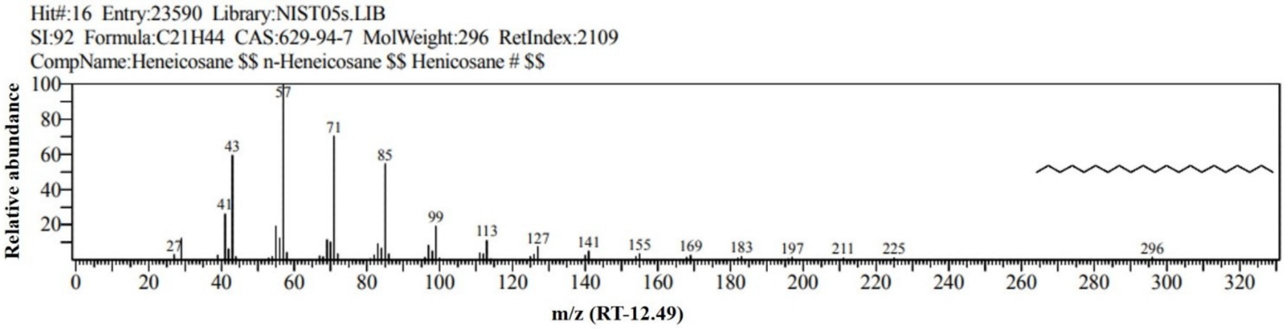

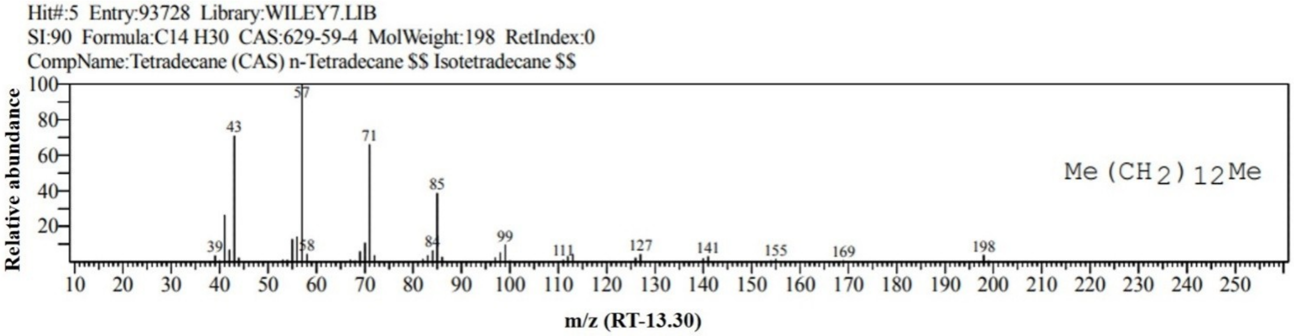

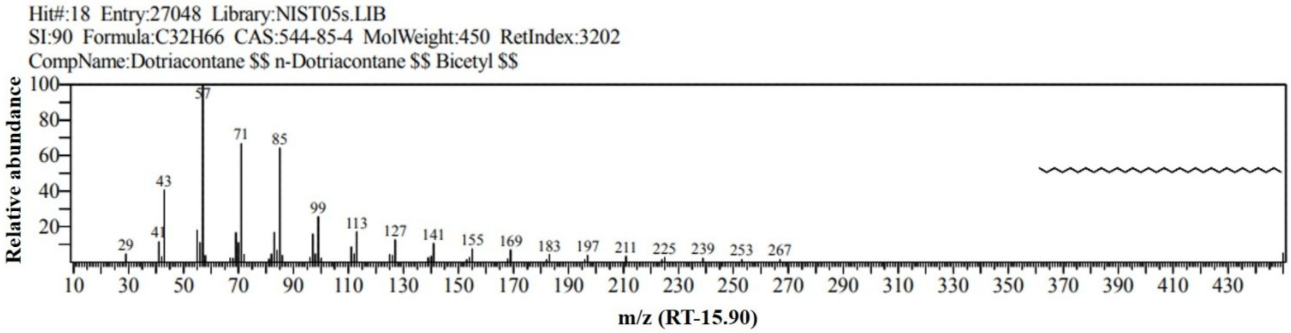

Supplementary Fig. S2: Mass spectrum of antimicrobial compounds identified in water diffusible extract of *Bacillus amyloliquefaciens* 8SE-IF1 through GC-MS/MS

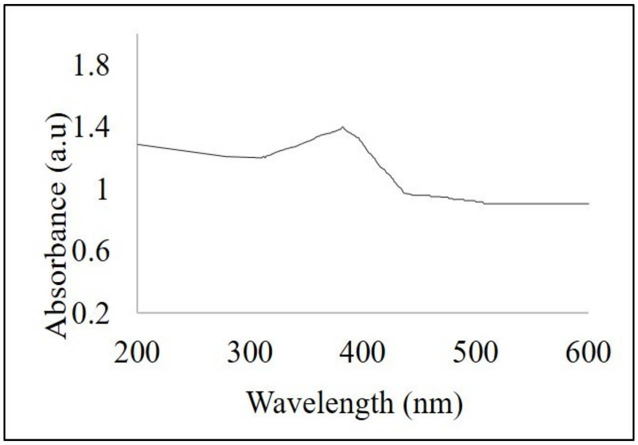

Supplementary Fig. S3: UV-Vis spectrum of green synthesized zinc oxide nanoparticles of water diffusible metabolites of *Bacillus amyloliquefaciens* 8SE-IF1
